# High-quality genome and variation map reveal valuable loci and the genetic basis of trait divergence driven by geographic dispersal in *Crotalaria pallida*

**DOI:** 10.1093/hr/uhag026

**Published:** 2026-01-29

**Authors:** Hubiao Yang, Xiaoxue Ye, Yiming Wang, Wei Yan, Changmian Ji, Yu Wang, Zehong Ding, Weiwei Tie, Fei Yan, Zhenfan Hao, Qian Liu, Zhengyang Zhong, Xuekui Dong, Ling Kang, Mufei Zhu, Hao Lv, Wei Hu, Guodao Liu, Zhibiao Nan

**Affiliations:** College of Pastoral Agriculture Science and Technology, Lanzhou University, Lanzhou, Gansu 730020, China; National Key Laboratory for Tropical Crop Breeding, Tropical Crops Genetic Resources Institute, Institute of Tropical Bioscience and Biotechnology, Sanya Research Institute, Chinese Academy of Tropical Agricultural Sciences, Haikou 571101, China; National Key Laboratory for Tropical Crop Breeding, Tropical Crops Genetic Resources Institute, Institute of Tropical Bioscience and Biotechnology, Sanya Research Institute, Chinese Academy of Tropical Agricultural Sciences, Haikou 571101, China; Hainan Key Laboratory for Protection and Utilization of Tropical Bioresources, Hainan Institute for Tropical Agricultural Resources, Chinese Academy of Tropical Agricultural Sciences, Haikou, Hainan 571101, China; Hainan Key Laboratory for Biosafety Monitoring and Molecular Breeding in Off-Season Reproduction Regions, Key Laboratory of Biology and Genetic Resources of Tropical Crops, Chinese Academy of Tropical Agricultural Sciences, Haikou, Hainan 571101, China; Novogene Bioinformatics Institute, Beijing 100000, China; Institute of Tropical and Subtropical Cash Crops, Yunnan Academy of Agricultural Sciences, Yunnan 678000, China; National Key Laboratory for Tropical Crop Breeding, Tropical Crops Genetic Resources Institute, Institute of Tropical Bioscience and Biotechnology, Sanya Research Institute, Chinese Academy of Tropical Agricultural Sciences, Haikou 571101, China; Hainan Key Laboratory for Protection and Utilization of Tropical Bioresources, Hainan Institute for Tropical Agricultural Resources, Chinese Academy of Tropical Agricultural Sciences, Haikou, Hainan 571101, China; Hainan Key Laboratory for Biosafety Monitoring and Molecular Breeding in Off-Season Reproduction Regions, Key Laboratory of Biology and Genetic Resources of Tropical Crops, Chinese Academy of Tropical Agricultural Sciences, Haikou, Hainan 571101, China; National Key Laboratory for Tropical Crop Breeding, Tropical Crops Genetic Resources Institute, Institute of Tropical Bioscience and Biotechnology, Sanya Research Institute, Chinese Academy of Tropical Agricultural Sciences, Haikou 571101, China; Hainan Key Laboratory for Protection and Utilization of Tropical Bioresources, Hainan Institute for Tropical Agricultural Resources, Chinese Academy of Tropical Agricultural Sciences, Haikou, Hainan 571101, China; Hainan Key Laboratory for Biosafety Monitoring and Molecular Breeding in Off-Season Reproduction Regions, Key Laboratory of Biology and Genetic Resources of Tropical Crops, Chinese Academy of Tropical Agricultural Sciences, Haikou, Hainan 571101, China; National Key Laboratory for Tropical Crop Breeding, Tropical Crops Genetic Resources Institute, Institute of Tropical Bioscience and Biotechnology, Sanya Research Institute, Chinese Academy of Tropical Agricultural Sciences, Haikou 571101, China; Hainan Key Laboratory for Protection and Utilization of Tropical Bioresources, Hainan Institute for Tropical Agricultural Resources, Chinese Academy of Tropical Agricultural Sciences, Haikou, Hainan 571101, China; Hainan Key Laboratory for Biosafety Monitoring and Molecular Breeding in Off-Season Reproduction Regions, Key Laboratory of Biology and Genetic Resources of Tropical Crops, Chinese Academy of Tropical Agricultural Sciences, Haikou, Hainan 571101, China; National Key Laboratory for Tropical Crop Breeding, Tropical Crops Genetic Resources Institute, Institute of Tropical Bioscience and Biotechnology, Sanya Research Institute, Chinese Academy of Tropical Agricultural Sciences, Haikou 571101, China; Hainan Key Laboratory for Protection and Utilization of Tropical Bioresources, Hainan Institute for Tropical Agricultural Resources, Chinese Academy of Tropical Agricultural Sciences, Haikou, Hainan 571101, China; Hainan Key Laboratory for Biosafety Monitoring and Molecular Breeding in Off-Season Reproduction Regions, Key Laboratory of Biology and Genetic Resources of Tropical Crops, Chinese Academy of Tropical Agricultural Sciences, Haikou, Hainan 571101, China; Shaanxi Provincial Bioresource Key Laboratory, College of Biological Science and Engineering, Shaanxi University of Technology, Hanzhong 723000, China; Mountains Bioresources Comprehensive Development C.I.C., Hanzhong 723001, China; College of Tropical Crops, Hainan University, Hainan 570228, China; Institute of Tropical and Subtropical Cash Crops, Yunnan Academy of Agricultural Sciences, Yunnan 678000, China; Wenshan Academy of Agricultural Sciences, Yunnan 663000, China; Wuhan Healthcare Metabolic Biotechnology Co., Ltd, Hubei 430000, China; Novogene Bioinformatics Institute, Beijing 100000, China; Novogene Bioinformatics Institute, Beijing 100000, China; Novogene Bioinformatics Institute, Beijing 100000, China; National Key Laboratory for Tropical Crop Breeding, Tropical Crops Genetic Resources Institute, Institute of Tropical Bioscience and Biotechnology, Sanya Research Institute, Chinese Academy of Tropical Agricultural Sciences, Haikou 571101, China; Hainan Key Laboratory for Protection and Utilization of Tropical Bioresources, Hainan Institute for Tropical Agricultural Resources, Chinese Academy of Tropical Agricultural Sciences, Haikou, Hainan 571101, China; Hainan Key Laboratory for Biosafety Monitoring and Molecular Breeding in Off-Season Reproduction Regions, Key Laboratory of Biology and Genetic Resources of Tropical Crops, Chinese Academy of Tropical Agricultural Sciences, Haikou, Hainan 571101, China; National Key Laboratory for Tropical Crop Breeding, Tropical Crops Genetic Resources Institute, Institute of Tropical Bioscience and Biotechnology, Sanya Research Institute, Chinese Academy of Tropical Agricultural Sciences, Haikou 571101, China; College of Pastoral Agriculture Science and Technology, Lanzhou University, Lanzhou, Gansu 730020, China

## Abstract

*Crotalaria* is a genus of the Fabaceae family with agricultural and medicinal value, but to date the genome has not been fully sequenced. Although *Crotalaria pallida* is widely distributed in tropical and subtropical regions, the degree of genetic diversity and the specific traits influenced by geographic dispersal remain unknown. We here report a high-quality genome assembly of *C. pallida* with 98.52% coverage which is assembled into 8 chromosomes. *C. pallida* is closely related to *Lupinus angustifolius*, with genetic divergence occurring ~42.5–57.4 million years ago (MYA). Re-sequencing of 236 *C. pallida* accessions revealed a genetic diversity decrease as *C. pallida* spread from Africa to America and Asia, and from Asia to China and finally to Hainan. Significant divergence was observed in seven traits between non-Hainan and Hainan accessions. Genome-wide association studies identified 73 loci for 18 agronomic traits, 25 of which overlapped with divergent sweeps between non-Hainan accessions and Hainan accessions. Furthermore, the dispersal of *C. pallida* in Hainan reduced genetic diversity, leading to a divergence in allelic frequencies at four candidate genes (*CpPTR*, *CpMYB*, *CpRLPK*, and *CpNADK*) associated with plant height. This study reveals the genetic basis of trait divergence driven by geographic dispersal and offers valuable resources for the strategic development of *C. pallida* breeding.

## Introduction

*Crotalaria* is a genus of the Fabaceae family comprising 702 species worldwide. The primary centers of *Crotalaria* diversity are tropical and subtropical Africa and Madagascar, where 543 species have been identified. Species of *Crotalaria* are also widely distributed in temperate and tropical Asia, South America, North America, and Australasia [1]. *Crotalaria* species include erect shrubs, as well as annual or short-lived perennial herbs with various uses, representing an agriculturally and medicinally important genus of the Fabaceae family [2–4]. However, research on the genomics and genetics of *Crotalaria* lags significantly behind that of other Fabaceae genera such as *Glycine*, *Cajanus,* and *Cicer* [5–7]. To date, no information is available on the whole genomes of *Crotalaria* genus, which greatly hinders efficient evaluation and utilization of germplasm resources, genetic improvement, and design of breeding strategies.

*Crotalaria pallida* is a species in the genus of *Crotalaria* with agricultural and medicinal value. Agriculturally, it can be used as green manure to reduce fertilizer application [8] as it is effective in nitrogen fixation and in increasing nutrient availability and organic matter in soil. It can also serve as a cover plant suppressing weed growth through allelopathy or competition. Additionally, it is an important source of fibers, paper pulp, silage, and diet for livestock [2, 3]. Compounds such as alkaloids and flavonoids with anti-inflammatory and antimicrobial effects in the roots, stems, leaves, and seeds of *C. pallida* have found therapeutic use in treating tumors, tinea scabies, rheumatoid arthritis, and traumatic injuries [9].

*Crotalaria pallida* is thought to have been introduced to the New World from Africa around 500 years ago [10]. It subsequently spread to many tropical, neotropical, and subtropical regions [2], with a high density from southern Brazil to the southeastern USA [10]. However, its geographical dispersal routes, genetic diversity, and the genetic basis of key traits remain poorly understood. Notably, although *C. pallida* is widely distributed, changes in genetic diversity and traits driven by geographic dispersal have yet to be systematically investigated.

In this study, we conducted high-quality genome assembly and large-scale re-sequencing of *C. pallida* accessions to explore their geographic dispersal routes, genetic diversity, and the genetic basis of key traits. We identified valuable loci associated with key traits and allelic variations in candidate genes, providing insights into the genetic basis of trait divergence of *C. pallida* driven by geographic dispersal. These data provide a resource for genetic studies of *Crotalaria* species and genomics-enabled improvements of *C. pallida*.

## Results

### Genome assembly and annotation

**Figure 1:**
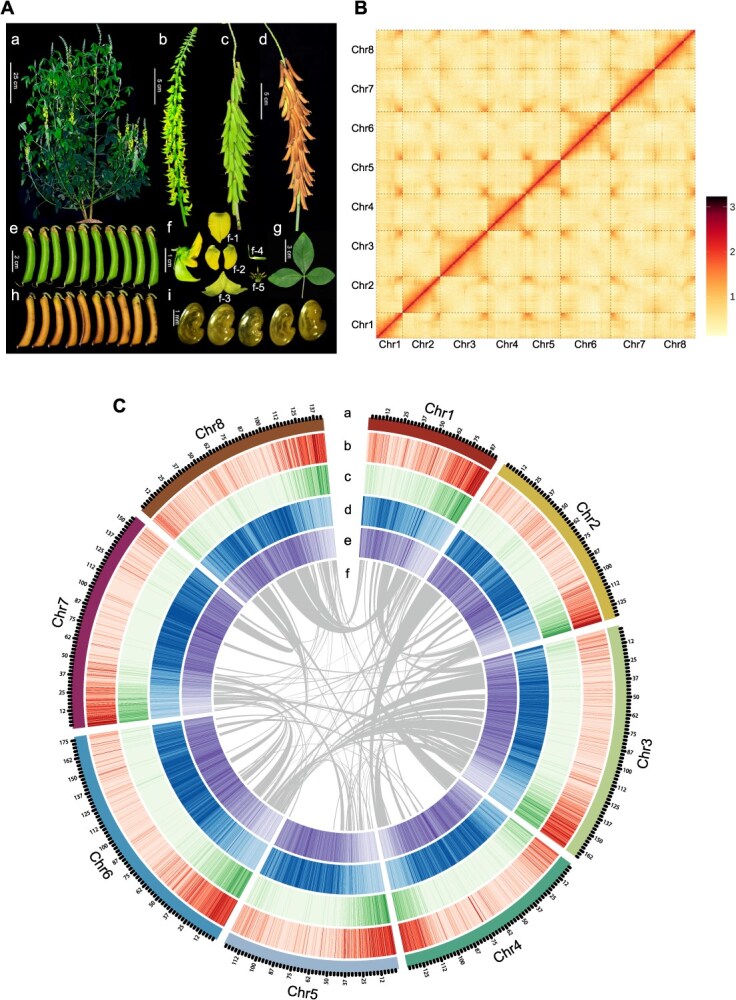
*C. pallida* genomic features. (A) Schematic presentation of *C. pallida.* a, *C. pallida* plant; b, inflorescence; c, infructescence (immature stage); d, infructescence (mature stage); e, immature pods; f, flower (f-1, standard; f-2, wings; f-3, Keel; f-4, Ovary; f-5, stamens); g, leaf; h, mature pods; i, seed. (B) Hi-C intrachromosomal contact map for each chromosome. Darker red indicates the high contact probability. (C) Distribution of genomic features. a, chromosomes; b, gene density; c, coding sequence density; d, repetitive sequence density; e, GC content; f, syntenic connections.

We assembled the *C. pallida* (C103_105 accession) genome using 58.9 Gb (42.5×) Illumina short reads, 225.0 Gb (162.4×) PacBio long reads, and 145.4 Gb chromosome conformation capture (Hi-C) pair-end reads (105.0×) (Fig. 1A and Table S1), producing a 1153.05-Mb assembled genome that covers 99.59% of the estimated genome size (Fig. S1). The assembly consisted of 252 scaffolds, with a contig N50 of 12.05 Mb and a scaffold N50 of 144.18 Mb (Table S2). Karyotype analysis showed 8 pairs of chromosomes (2*n* = 16) in *C. pallida* (Fig. S2). We anchored 1135.96 Mb (98.52%) of the assembly (1153.05 Mb oriented) and 98.89% of protein-coding genes onto eight pseudo-chromosomes using Hi-C links (Fig. 1B and Table S3). The assembly has 93% of the 1440 single-copy conserved orthologous genes in the BUSCO Embryophyta gene set (Table S4). We observed 14 telomeric tracks in the genome assembly (Table S5). The single-molecule long reads, Illumina short reads, and RNA-seq reads were properly remapped against the assembled genome (Tables S6 and S7). Together, these results demonstrated a high-quality assembly of the *C. pallida* genome.

The genome comprised 69.53% repetitive sequences, with LTR-retrotransposons representing the most abundant class (Gypsy elements: 37.36%; Copia elements: 16.62%). These elements tended to be enriched near the centromeric and pericentromeric regions (Fig. 1C, Fig. S3 and Table S8). Active insertions of LTR-retrotransposons occurred within 0.4 to 0.6 million years ago (MYA), representing 15.35% (5477 out of 35 688) of intact LTR-retrotransposons (Fig. S4).

We used homologous protein sequences, *ab initio* predictions, and transcriptomes from 27 samples to annotate the genome, and identified 31 762 protein-coding genes (Tables S9–S12). Of these, 31 410 genes were anchored onto the 8 pseudo-chromosomes, predominantly in chromosomal arms, and 80.14% were supported by RNA-Seq data (Fig. 1C, Table S3 and Figs S5–S7). We identified 7885 pseudogenes, 5624 non-coding RNAs, and 2298 transcription factors among 93 families (Tables S9 and S13).

### Comparative genomics and evolutionary analysis

We constructed a phylogenetic tree based on 419 single-copy orthologous genes shared among 14 species using *Arabidopsis thaliana* and *Vitis vinifera* as outgroups. We found that *C. pallida* has a relatively slower evolutionary rate compared with other legume species and is most closely related to *Lupinus angustifolius* (Fig. S8). The divergence between *C. pallida* and *L. angustifolius* occurred ~42.5 to 57.4 MYA, with their common ancestor having diverged at ~64.7 MYA (Fig. S9).

Using 13 additional plant genomes for comparison, we performed gene family clustering and expansion/contraction analyses on the *C. pallida* genome. Among the 14 plant genomes, 419 single-copy gene families were identified as conserved. (Fig. S10). In contrast, 799 gene families (containing 2384 genes) were specific to *C. pallida*. Additionally, gene family evolution analysis revealed 110 expanded families (4885 genes) and 68 contracted families (579 genes) in *C. pallida* (Fig. S11). Functional enrichment analysis showed that expanded families were significantly associated with homologous recombination, flavonoid biosynthesis, and anthocyanin biosynthesis pathways (Fig. S12 and Table S14).

The analysis of four-fold degenerate synonymous sites at the third codon positions (4DTv) across all gene pairs in the *C. pallida* genome revealed two prominent peaks. The first peak, around 0.24, suggested a whole-genome duplication event common to the Fabaceae family, while the second peak, near 0.65, pointed to the core eudicot γ triplication event (Fig. S13). Synteny analysis further indicated that *C. pallida* did not undergo a lineage-specific recent whole genome triplication (WGT), which is observed in the lupin clade [11] (Fig. S14).

### Flavonoids biosynthesis

Flavonoids are the primary bioactive constituents in medicinal plants [12, 13], and flavonoid biosynthesis was found to be enriched among expanded gene families in *C. pallida* (Fig. S12 and Table S14). Therefore, we collected leaves from *C. pallida* (accession C103_105) for targeted metabolomic analysis to quantify flavonoid metabolites. A total of 41 flavonoid metabolites were identified, among which the top five most abundant ones are cynaroside (luteolin-7-O-glucoside), diosmin, vitexin, orientin, and epimedin A (Table S15). Cynaroside exhibits antioxidant and anti-inflammatory activities [14]. Diosmin is a clinically validated pharmaceutical ingredient, widely used for the treatment of chronic venous insufficiency, hemorrhoids, lymphedema, and other related conditions [15]. Vitexin and orientin are isomers with potent antioxidant, radioprotective, anti-inflammatory, and neuroprotective properties [16]. Epimedin A exerts kidney-tonifying and yang-invigorating effects (a traditional medicinal activity) and promotes osteoblast proliferation [17]. These flavonoid metabolites are the principal medicinal components of *C. pallida*, offering deeper insights into its medicinal properties.

**Figure 2:**
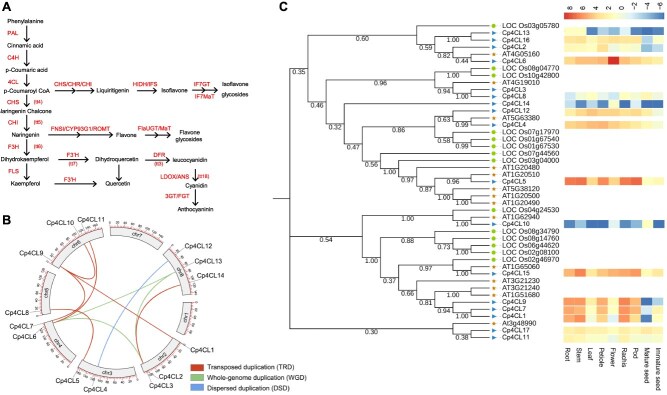
Flavonoid biosynthesis in *C. pallida*. (A) The flavonoid biosynthesis pathway. Key enzymes involved in the pathway are indicated, and their annotations are shown in Table S16. (B) Duplications of *Cp4CLs*. (C) Phylogenic relationship and expression of *Cp4CLs*. Expression of *Cp4CLs* was showed by a heatmap using RNA-seq data. Each sample contains three biological replicates.

Furthermore, transcriptome sequencing was performed to analyze the gene expression involved in flavonoid biosynthesis across various tissues. We totally annotated 166 flavonoid biosynthesis related genes in *C. pallida* genome and detected their expression among tissues (Fig. 2A and Table S16). These genes were further grouped into eight clusters for preliminary classification (Fig. S16) and showed tissue-specific expression patterns (Fig. S17 and Table S16). There were 17 genes encoding 4-coumarate-CoA ligase (4CL) in *C. pallida*, representing a notable expansion relative to other legume and non-legume species examined (Table S17). The 4CLs catalyze the ligation of coenzyme A with cinnamic acid as an important precursor for the biosynthesis of many flavonoids [18]. The expansion of *Cp4CLs* was driven by transposed duplication (TRD), whole-genome duplication (WGD), and dispersed duplication (DSD) (Fig. 2B). *Cp4CL4* and *Cp4CL12,* duplicated from DSD, showed a close phylogenic relationship and consistent expression patterns, with preferential expression in vegetative tissues (roots, stem, leaf, petiole, flower, rachis, pod) and low expression in seeds. *Cp4CL1, Cp4CL7,* and *Cp4CL9,* duplicated from TRD, also showed a close phylogenic relationship and similar expression patterns, with high expression levels in roots, stems and rachis. In contrast, *Cp4CL6* and *Cp4CL11*, *Cp4CL5* and *Cp4CL8*, and *Cp4CL9* and *Cp4CL10,* duplicated from TRD, showed a distant phylogenic relationship and differential expression. Additionally, *Cp4CL2, Cp4CL6, Cp4CL3* duplicated via WGD, shared close phylogenetic relationships but showed differential expression (Fig. 2B, C and Table S16). These results indicate that the expansion and evolution of *Cp4CLs* contribute to both conserved and variable expression, thereby facilitating flavonoids biosynthesis in *C. pallida.*

### Genome variation map, phylogeny, and genetic diversity

**Figure 3:**
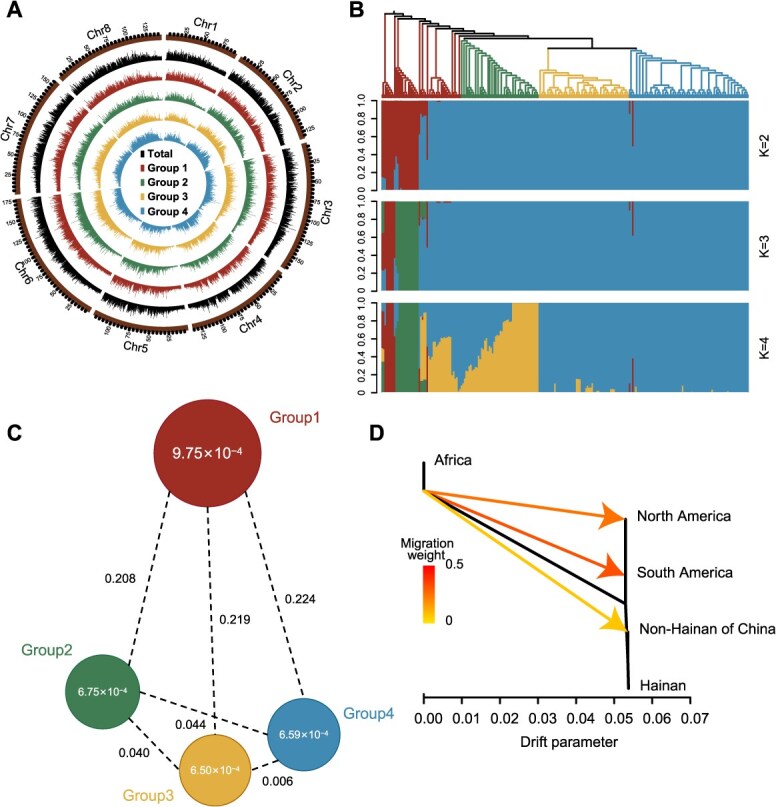
Phylogenetic relationships and genetic diversity of resequenced *C. pallida* accessions. (A) SNP density distributions of resequenced accessions. (B) Neighbour-joining phylogenetic tree and model-based clustering of resequenced accessions. Branches in the tree represent different groups, consistent with the grouping shown in A. (C) Genetic diversity (*θπ*) and population divergence (*F*_ST_) across the four groups. Circle values represent within-group *θπ*, and line values indicate pairwise *F*_ST_. (D) Gene flow of *C. pallida* accessions. Arrows indicate the directions of gene flow and lines represent the migration weights of accessions. Black slashes represent the branches of the phylogenetic tree. Branch lengths are scaled to present the magnitude of genetic drift between groups, defined by the drift parameter in the coalescent model.

A genome-wide variation map was constructed using 236 wild *C. pallida* accessions collected from 22 countries (Table S18). We generated 4.14 Tb of resequencing data, achieving an average sequencing depth of 14.07× and a genome coverage of 86.59% of the reference genome assembled in this study. In total, 5 319 493 high-quality single-nucleotide polymorphisms (SNPs) were detected, including 73 510 nonsynonymous mutations and 66 478 synonymous mutations. In addition, we identified 2 476 056 insertions and deletions (InDels), 4829 and 3252 InDels of which caused frameshift deletions and insertions, respectively (Fig. 3A and Table S19). This integrated variation data set represents a new resource for *C. pallida* genetics and breeding.

Phylogenetic and population-structure analyses divided these accessions into four cluters, designated as Groups 1, 2, 3, and 4, containing 51, 50, 58, and 77 accessions, respectively. Group 1 covered accessions from all five continents, primarily Asia (22 samples), South America (14 samples), and Africa (11 samples). Group 2 primarily included accessions from Hainan province (37 samples) of China. Group 3 mainly contained accessions from five other Chinese provinces (56 samples). Group 4 primarily included accessions from Hainan (46 samples) and Yunnan (15 samples) provinces of China (Fig. 3B and Fig. S18). Group 1 had a greater admixture in genetic composition and higher genetic diversity (*θπ* = 9.75 × 10^−4^) than the Group 2 (*θπ* = 6.75 × 10^−4^), Group 3 (*θπ* = 6.50 × 10^−4^), and Group 4 (*θπ* = 6.59 × 10^−4^). Accordingly, the *F*_ST_ value between Group1 and Groups 2–4 (0.208–0.224) was significantly higher than that between Group 2 and Group 3 (0.040), between Group 2 and Group 4 (0.044), and between Group 3 and Group 4 (0.006) (Fig. 3B and C).

Based on the geographic distribution, we found that genetic diversity was reduced in accessions from Africa (*θπ* = 8.34 × 10^−4^), North America (*θπ* = 8.02 × 10^−4^), South America (*θπ* = 7.94 × 10^−4^), Asia (*θπ* = 7.76 × 10^−4^), non-Hainan of China (*θπ* = 7.50 × 10^−4^), and Hainan Province of China (*θπ* = 6.96 × 10^−4^). Moreover, gene flow of *C. pallida* accessions was observed to be from Africa to North America, South America, and non-Hainan areas of China (Fig. 3D). These results indicate a possible dispersal of *C. pallida* from Africa to North America, South America, and China, supporting the hypothesis that *C. pallida* was introduced to the New World from Africa [10]. Further analyses of the demographic scenarios showed that the divergence of *C. pallida* from Africa to the America and Asia (Scenarios 1 and 2, Fig. S19), and the stepwise dispersal of *C. pallida* from non-Chinese Asian regions to non-Hainan areas of China, and finally to Hainan (Scenario 1; Fig. S20), were the most likely evolutionary scenarios.

### Genome-wide association study

Based on the 5 319 493 high-quality SNPs identified from 236 *C. pallida* accessions, we conducted a genome-wide association study (GWAS) for 30 morphological traits at two locations (Danzhou, Hainan Province, and Baoshan, Yunnan Province, China) in 2021 and 2022 (Table S20). We employed a mixed linear model (MLM) using GEMMA software [19], using the top three principal components (PCs) as fixed covariates and a kinship matrix as a random effect to control for population structure and cryptic relatedness, thereby reducing potential spurious associations. In total, there were 73 marker-trait associations (MTAs) identified for 18 key morphological traits (Table S21 and Fig. S21), with eight pairs of MTAs being repeatedly observed across different environments (Table S22). Given the agronomic importance of stem branches number, inflorescence length, and plant height traits that contribute to overall plant architecture affecting productivity [20, 21], we highlight several candidate genes putatively associated with these traits features below.

#### Number of stem branches

**Figure 4:**
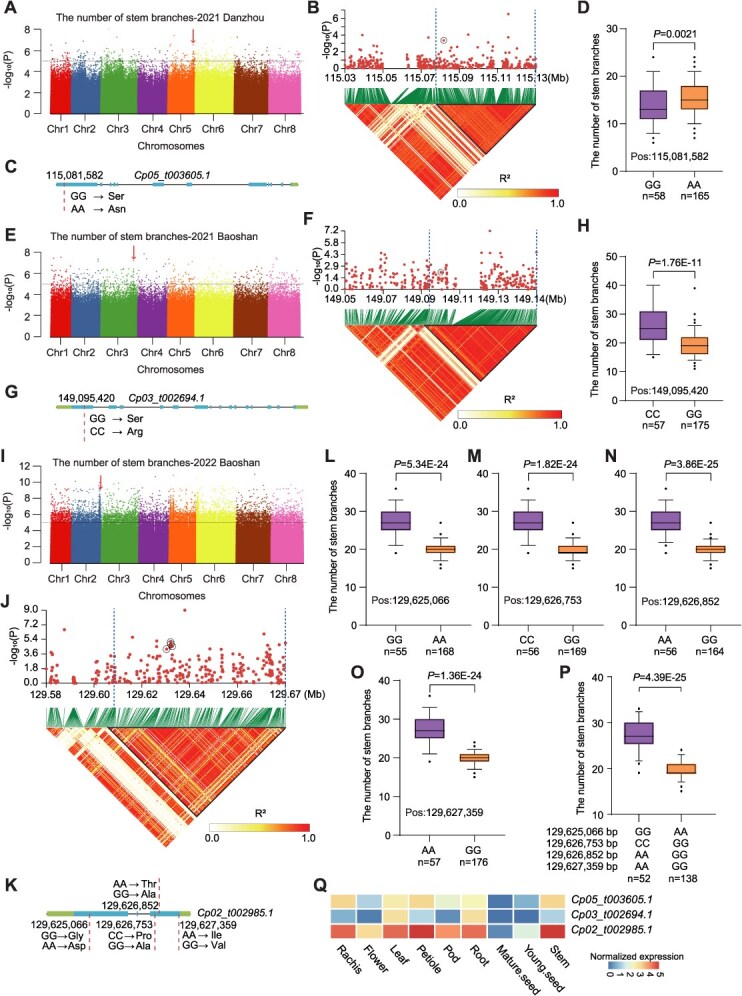
GWAS identification of candidate genes for the number of stem branches. (A–D) GWAS identification of *Cp05_t003605.1* as a candidate gene for the number of stem branches located on chromosome 5. (A) Manhattan plots displaying the GWAS results for the number of stem branches, with the significant GWAS peak indicated by an arrow. (B) Local Manhattan plot (top) and LD heat map (bottom) around the GWAS signal. Dashed lines represent the candidate region, and the core SNP in the candidate gene is circled. (C) *Cp05_t003605.1* gene model, with the position of nonsynonymous SNPs marked by a dashed line. (D) Comparison of the number of stem branches according to the core SNP variants in the candidate gene. (E–H) GWAS identification of *Cp03_t002694.1* as a candidate gene for the number of stem branches on chromosome 3. (I–P) GWAS identification of *Cp02_t002985.1* as a candidate gene for the number of stem branches on chromosome 2. Box plot annotations: Central line: Median value, Box limits: 25th and 75th percentiles (interquartile range), Whiskers: Data range within 1.5 × IQR (outliers plotted as individual points). Group differences were evaluated using two-tailed *t* test, with significance denoted as *P* < 0.05. (Q) Heatmap of the expression of three candidate genes across different tissues.

The *Cp05_t003605.1* near a signal (Chr 5: 115113158 bp) associated with the number of stem branches encodes a lysine-specific demethylase that is known to regulate brassinosteroid response (Fig. 4A and B) [22]. We found that a nonsynonymous SNP (Chr 5: 115081582 bp) within *Cp05_t003605.1* is significantly associated with stem branch number (Fig. 4C and D), a strong signal (Chr 3: 149120467 bp) is associated with the number of stem branches harbors *Cp03_t002694.1* (Fig. 4E and F), which encodes a Raf-like Ser/Thr protein kinase involved in tillering [23]; a nonsynonymous SNP (Chr3: 149095420 bp) in *Cp03_t002694.1* is associated with the number of stem branches (Fig. 4G–H); a significant peak (Chr 2: 129632570 bp) is associated with the number of stem branches contains *Cp02_t002985.1* (Fig. 4I and J)*,* which encodes an F-box protein that is crucial for strigolactone signaling and tillering [24–26] and, four nonsynonymous SNPs (Chr 2: 129625066 bp, 129 626 753 bp, 129 626 852 bp, and 129 627 359 bp) in *Cp02_t002985.1* are associated with the number of stem branches (Fig. 4K–O). Accessions harboring the GG-CC-AA-AA haplotype had a greater number of stem branches than those harboring the AA-GG-GG-GG haplotype (Fig. 4P). *Cp05_t003605.1* and *Cp02_t002985.1* are mainly expressed in the stem and petiole, whereas *Cp03_t002694.1* shows relatively low expression, primarily detected in the leaf and root (Fig. 4Q**,**  Table S23).

#### Inflorescence length

An inflorescence length signal (Chr 3: 162275062 bp) was detected in the fifth exon of *Cp03_t003762.1*, which encodes an MYB transcription factor involved in the formation of meristems [27]. A nonsynonymous SNP (Chr 3: 162275062 bp) in *Cp03_t003762.1* is associated with inflorescence length (Fig. S22). *Cp03_t003762.1* is mainly expressed in flowers (Table S23).

#### Plant height

The *Cp02_t002910.1* near a significant signal (Chr 2: 128691294 bp) associated with plant height 80 days after planting encodes a B3 domain-containing protein that regulates seedling development by affecting carbohydrate and gibberellin metabolism (Fig. S23A and B) [28]. Two nonsynonymous SNPs (Chr 2: 128675433 bp and 128 676 692 bp) in *Cp02_t002910.1* are associated with plant height (Fig. S23C and D). Accessions harboring the TT-AA haplotype had higher plant height than those harboring the GG-TT haplotype (Fig. S23E). Another significant signal on chromosome 5 (Chr 5: 7801251 bp) associated with plant height 80 days after planting harbors *Cp05_t000725.1* (Fig. S23F–G), which encodes a bHLH transcription factor involved in regulating internode elongation and plant height [29, 30]. A nonsynonymous SNP at Chr 5: 7761859 bp within *Cp05_t000725.1* is associated with plant height (Fig. S23H and I). *Cp02_t002910.1* is mainly expressed in mature seed, root and stem, while *Cp05_t000725.1* shows relatively high expression, primarily in the stem and root (Table S23).

Transcriptome analysis of candidate genes revealed their high expression across *C. pallida* tissues (Table S23). When combined with the previous allelic variation analysis, the MTAs identified in this study, along with the accessions containing advantageous alleles, would facilitate efficient germplasm resource utilization and accelerate *C. pallida* genetic improvement.

### Divergence between G1NH and G2H/G3H/G4H

**Figure 5:**
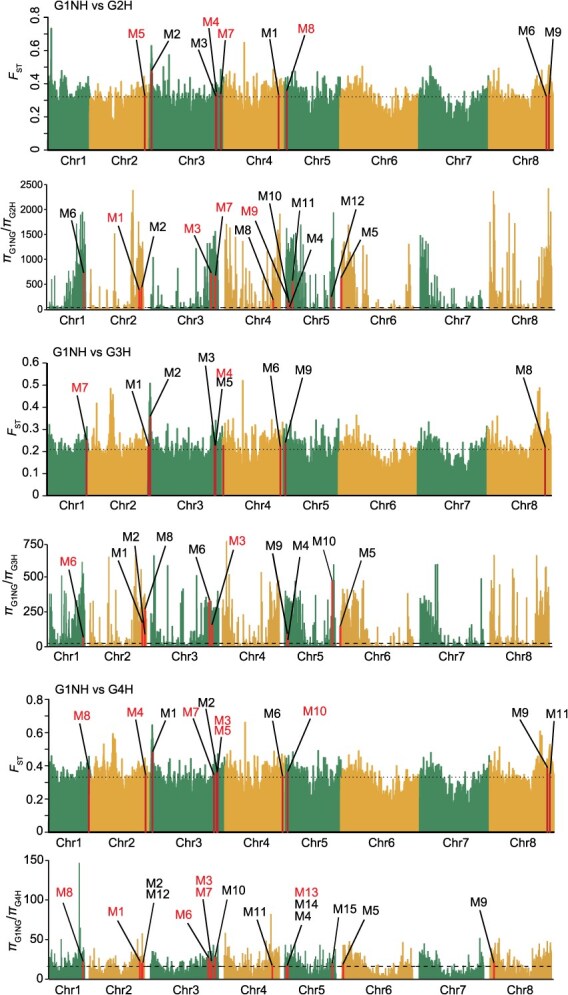
Divergent regions between G1NH and G2H/G3H/G4H and overlapping with GWAS signals. G1NH, Non-Hainan accessions in Group 1. G2H, Hainan accessions in Group 2. G3H, Hainan accessions in Group 3. G4H, Hainan accessions in Group 4. The horizontal black dashed lines indicate values above the 95% threshold for divergent sweeps. The black slant lines show the position of highly divergent regions overlapped with GWAS peaks. The detailed information is shown in Tables S35–S40. Divergent genomic regions identified by the two methods are highlighted.

Since Group 1 showed an obvious admixture in genetic composition and high genetic diversity, whereas Groups 2, 3, and 4 had low genetic diversity, in particular for those accessions from Hainan (Fig. 3B and C), we used pairwise fixation statistic (*F*_ST_) analysis to identify the significantly divergent sweeps between G1NH (Non-Hainan accessions in Group 1, 44 accessions) and G2H (Hainan accessions in Group 2, 37 accessions), between G1NH and G3H (Hainan accessions in Group 3, 9 accessions), and between G1NH and G4H (Hainan accessions in Group 4, 46 accessions). A total of 1531, 1447, and 1545 genomic regions with significant genetic divergence (top 5% of *F*_ST_ values) in G1NH versus G2H, G1NH versus G3H, and G1NH versus G4H, covering 5462, 4750, and 5492 genes, respectively (Fig. 5 and Tables S24–S26) were found. In addition, a total of 1111 divergent genomic regions (covering 3814 genes) were found to be conserved in G1NH versus G2H, G1NH versus G3H, and G1NH versus G4H (Table S27). Moreover, we found a total of 976, 951, and 1195 genomic regions with significant genetic divergence (top 5% of *F*_ST_ values) in G2H versus G3H, G2H versus G4H, and G3H versus G4H, covering 4192, 3799, and 2018 genes, respectively (Tables S28–S30).

We also analyzed nucleotide diversity (*π*) ratios to identify the regions with significantly decreased genetic diversity from G1NH to G2H, G3H, and G4H, respectively. We found a total of 1710, 1838, and 1703 genomic regions with significantly decreased genetic diversity (top 5% of *π* ratios) in G1NH versus G2H, G1NH versus G3H, and G1NH versus G4H, covering 7066, 6778, and 7297 genes, respectively (Fig. 5 and Tables S31–S33). In addition, a total of 881 divergent genomic regions (covering 5056 genes) were found to be conserved in G1NH versus G2H, G1NH versus G3H, and G1NH versus G4H (Table S34).

Since there was a significant trait divergence in plant height, stem branching, inflorescence length, and the number of flowers per inflorescence between G1NH and G2H/G3H/G4H (Fig. S24), we overlapped GWAS signals related to these traits with divergent genomic regions identified by *Fst*. The results showed that 9, 9, and 11 GWAS signals overlapped with divergent genomic regions in G1NH versus G2H, G1NH versus G3H, and G1NH versus G4H, respectively (Fig. 5 and Tables S35–S37). Several candidate genes associated with plant height under divergent sweeps were demonstrated as follows (Figs S25–S27). We also overlapped GWAS signals related to these traits with divergent genomic regions identified by *π*. The results showed that 12, 10, and 15 GWAS signals overlapped with divergent genomic regions in G1NH versus G2H, G1NH versus G3H, and G1NH versus G4H, respectively (Fig. 5 and Tables S38–S40). In total, 25 GWAS signals overlapped with divergent genomic regions in G1NH versus G2H/G3H/G4H identified by *Fst* or *π*. Among them, 12 divergent genomic regions were identified by both methods, overlapping with 7 MTAs (Table S41).

**Figure 6:**
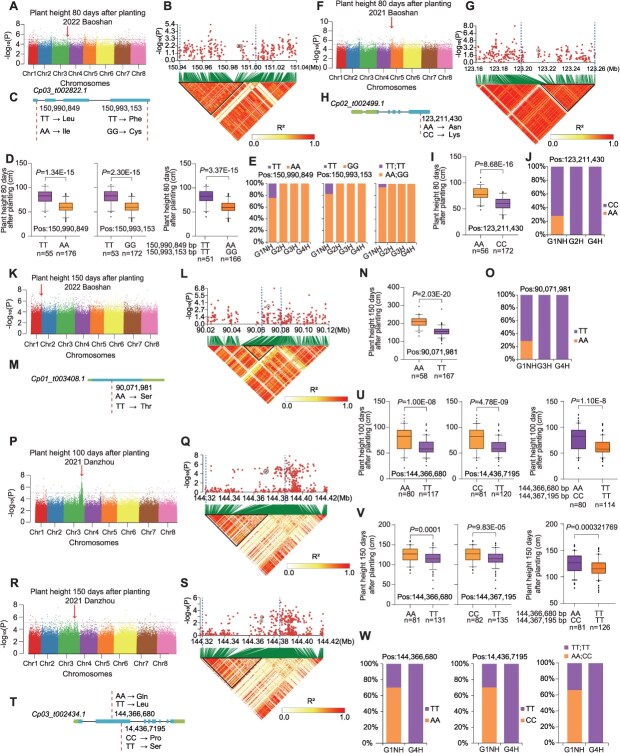
GWAS identification of plant height associated candidate genes that underwent divergence between G1NH and G2H/G3H/G4H. (A–E) GWAS identification of *Cp03_t002822.1* as a candidate gene for plant height 80 days after planting on chromosome 3. (A) Manhattan plots displaying the GWAS results for plant height measured 80 days after planting. An arrow highlights the significant GWAS peak. (B) A local Manhattan plot (top) and LD heat map (bottom) are shown around the GWAS signal. The candidate region is demarcated by dashed lines, and core SNPs within the candidate gene are circled. (C) *Cp03_t002822.1* gene model. The dashed lines indicate the position of nonsynonymous SNPs. (D) Comparison of plant height 80 days after planting based on the core SNP variants in the candidate gene. (E) Frequency of the core SNP variants in the candidate gene in G1NH (Non-Hainan accessions in group 1), G2H (Hainan accessions in Group 2), G3H (Hainan accessions in Group 3) and G4H (Hainan accessions in group 4). (F-J) GWAS identification of *Cp02_t002499.1* as a candidate gene for plant height 80 days after planting on chromosome 2. (K–O) GWAS identification of *Cp01_t003408.1* as a candidate gene for plant height 150 days after planting on chromosome 1. (P–W) GWAS identification of *Cp03_t002434.1* as a candidate gene for plant height 100 and 150 days after planting on chromosome 3. Box plot annotations: Central line: Median value, Box limits: 25th and 75th percentiles (interquartile range), Whiskers: Data range within 1.5 × IQR (outliers plotted as individual points). Group differences were evaluated using two-tailed *t*-test, with significance denoted as *P* < 0.05.

A significant signal (Chr 3: 150967314 bp) associated with plant height 80 days after planting harbors *CpPTR* (*Cp03_t002822.1*) (Fig. 6A and B), which encodes a PTR/NRT1 transporter involved in transporting plant hormones and increasing plant growth [31, 32]. Two nonsynonymous SNPs (Chr 3: 150990849 bp and 150 993 153 bp) in *CpPTR* are associated with plant height (Fig. 6C). The accessions harboring the TT allele (Chr 3: 150990849 bp) and TT allele (Chr 3: 150993153 bp) showed higher plant height than those harboring the AA allele (Chr 3: 150990849 bp) and the GG allele (Chr 3: 150993153 bp), respectively (Fig. 6D). This is in accordance with the divergence of the alleles frequency and plant height 80 days after planting between G1NH and G2H/G3H/G4H (Fig. 6E and Figs S24B and S25). It also showed decreased genetic diversity from G1NH to G2H and G4H (Tables S31 and S33). G1NH, which showed higher plant height than G2H/G3H/G4H were found to harbor the TT-TT haplotype (Fig. 6E).

Another significant signal (Chr 2: 123192660 bp) associated with plant height 80 days after planting was found to harbor *CpMYB* (*Cp02_t002499.1*) (Fig. 6F and G), which encodes an MYB transcription factor involved in regulating plant height [33]. A nonsynonymous SNP (Chr 2: 123211430 bp) in *CpMYB* was also found to be associated with plant height (Fig. 6H). A significant difference in plant height was observed between accessions with the AA and CC alleles, with the former showing greater height than the latter (Fig. 6I), which is in agreement with the divergence of the allele frequency and plant height 80 days after planting between G1NH and G2H/G4H (Fig. 6J and Figs S24B, S26). It also showed decreased genetic diversity from G1NH to G2H/G3H/G4H (Tables S31–S33). G1NH that showed higher plant height than G2H and G4H had the AA allele, whereas the AA allele was not found in G2H and G4H (Fig. 6J).

The *CpRLPK* (*Cp01_t003408.1*) near a significant signal (Chr 1: 90076758 bp) associated with plant height 150 days after planting encodes a receptor-like protein kinase involved in shoot growth (Fig. 6K–L) [34]. A nonsynonymous SNP (Chr 1: 90071981 bp) in *CpRLPK* is associated with plant height (Fig. 6M). The accessions carrying the AA allele exhibited significantly greater plant height than those harboring the TT allele (Fig. 6N). This is in accordance with the divergence of allele frequency and plant height 150 days after planting between G1NH and G3H/G4H (Fig. 6O and Figs S24G, S27). G1NH that showed higher plant height than G3H/G4H had the AA allele, whereas all of the accessions in G3H/G4H carried only the TT alleles (Fig. 6O).

Two strong peaks (Chr 3: 144386162 bp and 144 382 380 bp) associated with plant height 100 days and 150 days after planting commonly harbor *CpNADK* (*Cp03_t002434.1*) (Fig. 6P–S), which encodes a NAD kinase that is known as a growth repressor [35]. Two nonsynonymous SNPs (Chr 3: 144366680 bp and 144 367 195 bp) in *CpNADK* are associated with plant height 100 days and 150 days after planting (Fig. 6T). The accessions containing the AA allele (Chr 3: 144366680 bp) and CC allele (Chr 3: 144367195 bp) showed higher plant height than those containing the TT allele (Chr 3: 144366680 bp) and TT allele (Chr 3: 144367195 bp), respectively (Fig. 6U and V). This is in agreement with the divergence of alleles frequency and plant height 100 days and 150 days after planting between G1NH and G4H (Fig. 6W and Figs S24D–H, S25). It also showed decreased genetic diversity from G1NH to G3H and G4H (Tables S32 and S33). G1NH, which showed higher plant height than G4H, had the AA-CC haplotype, whereas all accessions in G4H only carried the TT-TT haplotype (Fig. 6W).

Together, these results indicated that decreased genetic diversity of the Hainan accessions resulted in divergence of allelic frequency in the candidate genes associated with plant height between G1NH and G2H/G3H/G4H, providing potential targets for selection aimed at modifying plant height.

## Discussion

*Crotalaria* spp. in the Fabaceae family are highly valued for their agricultural and medicinal uses [2, 3]. The assessment and exploitation of large-scale germplasm collections play a pivotal role in crop genetic enhancement, enabling the identification of genomic variations underlying key agronomic traits [5, 36]. However, the lack of a reference genome and whole genome variation information for the *Crotalaria* genus has greatly hampered the efficient evaluation, utilization of germplasm resources, genetic improvement, as well as the design of breeding strategies.

Here, we presented a high-quality genome assembly of *C. pallida*. *C. pallida* is most closely related to *L. angustifolius* with the divergence between *C. pallida* and *L. angustifolius* estimated to have occurred ~42.5 to 57.4 million years ago (MYA). The *C. pallida* genome underwent a WGD event shared by the Fabaceae family, as well as a core eudicot γ triplication event, consistent with genomes of *Glycine max*, *Astragalus sinicus* and *Medicago sativa* in Fabaceae family [37, 38]. However, it did not undergo a recent WGT that occurred in the lupin clade [11]. Gene duplication contributes to the generation of new genetic diversity and forms the basis for evolutionary innovation in eukaryotes [39, 40]. Gene families related to flavonoid biosynthesis were significantly expanded in *C. pallida,* with the expansion of *Cp4CLs* driven by WGD, transposed duplication (TRD), and DSD contributing to flavonoid biosynthesis. These genetic developments may assist in legume nodulation and the enhancement of biological activities for medicinal use [9, 38]. Together, these studies expand our knowledge of the genome evolution of *Crotalaria*, and provide insights into flavonoid biosynthesis of *C. pallida* from a genome perspective.

The genetic improvement of crops is further facilitated by evaluating germplasm resources and assessing allelic variations associated with agronomic traits [5]. To date, no study has utilized large-scale resequencing datasets to achieve fine-grained mapping of key agronomic trait loci in *Crotalaria*, a methodological limitation that our research aims to address. In this study, we identified 73 loci for 18 agronomic traits in *C. pallida* through GWAS analysis based on resequencing and phenotyping of 236 accessions. Among these, we identified 10 candidate loci associated with stem branching, inflorescence length, and plant height. The validity of these candidate genes is corroborated by two lines of evidence: allelic variation–phenotype associations in *Crotalaria*, and functional characterization of orthologous genes in model plant systems. This information could facilitate genetic improvement and the design of breeding strategies for *C. pallida*.

Four genetic groups were identified among *C. pallida* accessions through integrated phylogenetic and population structure analyses. Group 1 exhibited a clear admixture in genetic composition and high genetic diversity, whereas Groups 2, 3, and 4 showed low genetic diversity, particularly in accessions from Hainan. This is in agreement with the dispersal of *C. pallida* from Africa to America and Asia, and from Asia to non-Hainan areas of China and finally to Hainan. Interestingly, there was a significant divergence in plant height, stem branching, inflorescence length and the number of flowers per inflorescence between G1NH and G2H/G3H/G4H. By combining divergent sweeps and GWAS analyses, we revealed four candidate genes (encoding the PTR/NRT1 transporter, MYB transcription factor, receptor-like protein kinase and NAD kinase) associated with plant height. These genes underwent divergent sweeps between G1NH and G2H/G3H/G4H and exhibited progressive decreases in genetic diversity from G1NH to G2H/G3H/G4H. This finding is further supported by divergence in allelic frequency in the candidate genes, which is highly associated with divergence of plant height. These results indicate that geographic dispersal has influenced the genetic diversity of different groups, and has impacted the distribution and divergence of key traits in *C. pallida*.

**Figure 7:**
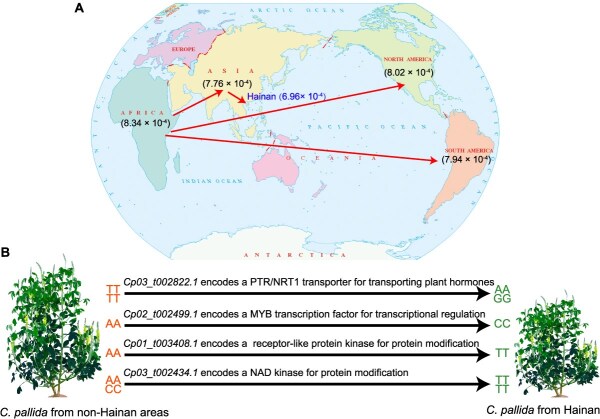
Proposed model for geographic dispersal affecting genetic diversity and the trait divergence of *C. pallida*. (A) Dispersal and genetic diversity of *C. pallida*. The number in parentheses indicates the value of genetic diversity *θπ*. Arrows indicate the direction of gene flow. (B) Allelic variation of candidate genes between accessions from non-Hainan areas in group 1 and accessions from Hainan in Groups 2, 3, and 4 affects plant height.

Building on these results, we formulate a model illustrating how geographic dispersal affects genetic diversity and traits in *C. pallida*. As *C. pallida* dispersed from Africa to America and Asia, and from Asia to China and finally to Hainan, its genetic diversity has decreased significantly (Fig. 7A). Compared to non-Hainan areas, the significant decrease in genetic diversity in *C. pallida* from Hainan has resulted in allelic variation in several candidate genes associated with plant height. The divergence in allelic frequency of these candidate genes, which are involved in hormone transport, transcriptional regulation, and protein modification by kinases, has resulted in reduced plant height in Hainan accessions, providing potential targets for breeding strategies aimed at modifying plant height (Fig. 7B).

In conclusion, this study presents a high-quality genome assembly and variation map for *C. pallida*, identified valuable loci underlying key agronomic traits, and provided genomic perspectives on trait divergence within *C. pallida* populations. Our findings elucidate the genetic architecture of geographic dispersal-driven trait divergence in *C. pallida* enabling the strategic exploitation of *C. pallida* for breeding. A rich repository of genomic resources for *C. pallida* is herein provided, enabling the research community to advance genetic improvement efforts and functional genomics studies in this important legume species.

## Materials and methods

### Genome sequencing

Genomic DNA was extracted from *Crotalaria pallida* (C103_105 accession) leaf tissue using the DNAsecure Plant Kit (TIANGEN, Beijing, China). The purified DNA was randomly fragmented, and short-reads libraries were prepared following the Illumina (San Diego, CA, USA) protocol and sequenced on the Illumina HiSeq X Ten platform. For long-read DNA sequencing, a 60 kb Single Molecule Real-Time (SMRT) long-reads library was prepared and sequenced using PacBio Sequel systems. The Hi-C library was prepared according to the standard procedure: nucleus DNA from *in vivo* cells of young leaves was first subjected to *in situ* cross-linked, followed by extraction and digestion with the restriction enzyme *Dpn*II for chromatin fragmentation. The sticky ends of digest fragments were labeled by biotin, diluted and randomly ligated to each other to form chimeric circles. Biotinylated fragments were subsequently enriched and further sheared as the sequencing library and sequenced on an Illumina HiSeq PE150 platform.

### Genome annotation

We identified transposable elements (TEs) through an integrated approach combining *de novo* prediction and homology-based methods. For the *de novo* detection, we employed RepeatModeler (version 1.0.4), LTR_Finder (version 1.0.7) [41], and RepeatScout (version 1.0.5). Classification and masking were performed using RepeatMasker (version 4.0.5) and RepeatProteinMask (version 4.0.5). Tandem repeats were annotated with Tandem Repeats Finder (version 4.07b) [42].

Protein-coding genes in the *C. pallida* genome were predicted using a comprehensive strategy incorporating three complementary approaches. For homolog-based prediction, proteins sequences from six plant species (*Cajanus cajan*, *Cicer arietinum*, *Lotus japonicus*, *Lupinus albus*, *Medicago truncatula*, and *Trifolium pratense*) were aligned to the genome using TblastN (*E*-value ≤ 1E − 5.), with gene models constructed using GeneWise (version 2.4.1) [43]. For *ab initio* prediction, five prediction programs were used to predict genes, namely, Augustus (version 3.2.3) [44], GENSCAN (version 1.0), geneid (version 1.4), GlimmerHMM (version 3.0.4) [45] and SNAP (version 2006-07-28) [46]. For transcriptome-based prediction, RNA-seq data from nine tissues (roots, stems, leaves, petioles, flowers, rachis, pods, immature seeds, and mature seeds) were mapped to the *C. pallida* genome using TopHat (version 2.0.13) [47] to identify exon regions and splice positions. Transcript assembly was performed with Cufflinks (version 2.1.1) using the alignment data, after which EVidenceModeler (version 1.1.1) [48] subsequently merged the different prediction sets while removing duplicate entries.

Protein-coding genes of the *C. pallida* were functionally annotated against public protein databases, including Swiss-Prot, NR, InterPro (version 32.0), Pfam (version 27.0), and KEGG. The tRNA genes were identified using tRNAscan-SE software (version 1.3.1) [49], while rRNA fragments were detected by BlastN (E-value ≤1E-10) against plant rRNA sequences. Infernal software (version 1.1rc4) [50] was used to predict miRNA and snRNA genes by screening the Rfam database (version 13.0).

### Comparative genomics analysis

OrthoMCL was used to construct orthologous gene families between the *C. pallida* and 13 other plant species (*Arabidopsis thaliana*, *Arachis duranensis*, *C. cajan*, *C. arietinum*, *Glycine max*, *L. japonicus*, *L. angustifolius*, *Medicago sativa*, *M. truncatula*, *Phaseolus vulgaris*, *T. pratense*, *Vigna angularis*, and *Vitis vinifera*) [51]. A set of 419 single-copy orthologs among 14 species was aligned using MUSCLE (version 3.7), and a maximum likelihood phylogenetic tree was constructed with RAxML software (version 8.0.19) under the PROTGAMMAAUTO model, with *V. vinifera* as an outgroup [52]. Divergence times were estimated using PAML v4.5's MCMCTree, utilizing CDS alignments derived from protein alignments [53]. Six calibration points from the TimeTree database were selected. The Expansion and contraction of orthologous gene families were assessed using CAFÉ (version e1.6). To investigate WGD events, syntenic region between and within *C. pallida*, *G. max*, *L. angustifolius*, *A. thaliana*, *M. truncatula*, and *A. duranensis* was carried out by MCScanX (version 0.8) based on the all-to-all BLASTP results [54]. Protein sequences of syntenic gene pairs were aligned with MUSCLE program, and 4DTv values were calculated based on the CDS alignments, with substitution rate corrections applied using the HKY model.

### Collection, extraction and detection of targeted flavonoid metabolome samples

Approximately 50 ± 2.5 mg of *C. pallida* (accession C103_105) leaf samples were weighed and homogenized with 600 μl of 70% methanol aqueous solution containing 0.1% hydrochloric acid. After vortexing (15 min) and ultrasonication (15 min), the mixture was centrifuged at 12000 rpm for 2 min. The supernatant was filtered and analyzed using an AB SCIEX QTRAP 6500+ LC–MS/MS system equipped with an ACQUITY HSS T3 column (2.1 × 100 mm, 1.8 μm) maintained at 40°C. The mobile phase consisted of 0.05% formic acid in water (A) and 0.05% formic acid in acetonitrile (B), with a flow rate of 0.35 ml/min. The gradient elution program was as follows: initial conditions of 90% A and 10% B were held for 1.5 min, followed by a linear change to 30% A by 7.5 min. The composition was then rapidly shifted to 95% B and held until 8.6 min for a column wash, before returning to the initial conditions (90% A) at 8.7 min for re-equilibration until the end of the 11-min run. The injection volume was 2 μl. Mass spectrometric detection was conducted in electrospray ionization (ESI) positive/negative modes (ion spray voltage: 5500 V/−4500 V) with a source temperature of 550°C. Curtain gas, ion source gas 1, and ion source gas 2 were set at 40, 50, and 60 psi, respectively. Data were acquired in Multiple Reaction Monitoring (MRM) mode with medium collision gas; entrance potential and collision cell exit potential were 10 V and −10 V for both polarities. Accurate quantification was achieved using a standard calibration curve (0.1–500 ng/ml, equivalent to 0.5–2500 nmol/l).

### RNA sequencing and expression analysis

Tissues samples of *C. pallida* were collected from nine organs (roots, stems, leaves, petioles, flowers, rachis, pods, immature seeds, and mature seeds), with triplicate RNA extractions from each tissue type using standardized extraction program. Following the manufacturer's protocol, we constructed cDNA libraries and sequenced them using the Illumina NovaSeq 6000 platform. The filtered reads were aligned to the *C. pallida* genome using HISAT2 (version 2.0.4) software with default parameters. The reads that mapped to genes were quantified using HTSeq (version 0.6.1p1) [55]. We identified differentially expressed genes (DEGs) using DESeq v1.10.1, defining significance as genes exhibiting an absolute fold change (FC) ≥ 2 and false discovery rate (FDR) < 0.05.

### Sampling and whole-genome re-sequencing

For each sample, high-quality DNA (1.5 μg) was sheared to construct the short-reads libraries (~350 bp insert size) following Illumina's standard protocol. Library quantification and integrity analysis were conducted using an Agilent 2100 Bioanalyzer, and whole genome sequencing was carried out on an Illumina NovaSeq 6000 platform.

### Reads mapping and SNP calling

We used BWA (version 0.7.8) to align the high quality paired-end reads to the assembled *C. pallida* reference genome with the parameter: ‘mem -t 4 -k 32 -M’ [56]. After sorting and deduplicating the bam files using SAMtools (version 0.1.19) command ‘sort’ and ‘rmdup’ [57], SNPs were called by using SAMtools (version 1.3.1) with the parameter: ‘-q 1 -C 50 -t SP -t DP -m 2 -F 0.002’ and BCFtools (version 1.3.1) with the parameter: ‘-Q 20 -d 2 -D 10000000’. The raw SNP dataset was filtered with a call rate ≥ 90%, a minor allele frequency (MAF) ≥ 1%, and supported by at least five mapped reads. The high-quality SNPs were identified and used in subsequent analyses.

### Annotation of genetic variants

Based on the *C. pallida* genome annotation, the SNPs were annotated using ANNOVAR (version 2013-05-09) [58]. These SNPs were grouped based on their genomic locations. Categories included exonic (overlapping with coding exons), intronic (within introns), splicing sites (within 2 bp of splice junction), 5′ and 3′ untranslated regions (UTRs), upstream and downstream regions (within 1 kb of region upstream of the transcription start or end site), and intergenic regions. SNPs located in coding exons were further classified as either synonymous or nonsynonymous.

### Population structure and phylogenetic analyses

To analyze the population structure, a neighbor-joining (NJ) tree was constructed using the program TreeBeST (version 1.9.2) with 1000 bootstrap replicates. The tree was displayed using iTOL. We investigated population structure using the program ADMIXTURE (version 1.23) [59]. To identify the best genetic cluster *K*, a cross-validation (CV) value was tested for each *K* values ranging from 2 to 8.

### Genetic diversity and selective sweep analyses

We assessed genetic nucleotide diversity using population genomic metrics, including nucleotide diversity (θπ, calculated as the average number of pairwise nucleotide differences per site) and population differentiation (*Fst*, Fixation index) within and between different groups. Analyses were performed in a 40 kb sliding window (20 kb step size) across the *C. pallida* genome using VCFtools (version 0.1.14) [60]. Regions under putative selection were identified as windows falling within the top 5% of *F*_ST_ scores.

### Gene flow analysis

Gene flow between *C. pallida* populations was estimated using TreeMix (version 1.13), which models population splits and admixture events. Continental populations (Asia, Africa, South America, and North America) were analyzed with the parameters: -bootstrap -k 100 -m, and values for -m ranging from 1 to 5. The allele frequencies for the TreeMix analysis were calculated by PLINK (version 1.07) software [61].

### Demographic history analysis

Four models representing demographic history of Africa, America, and Asia groups as well as non-China of Asia, non-Hainan of China, and Hainan groups were respectively evaluated using fastSimcoal3 (version 3.7.9). To ensure parameter convergence and robust likelihood estimation, we performed 100 independent runs of fastSimcoal3 for each model. Each run incorporated with 100 000 coalescent simulations (-n 100 000) to estimate likelihoods and 40 ECM cycles (-L 40) to iteratively improve parameter estimates. The optimal run for each model was identified by selecting the highest log-likelihood value, and the best overall model was chosen via maximum likelihood comparison for subsequent analyses. To better understand the differentiation of groups, we used Stairway Plot (version 2.1.1) software to estimate the historical *Ne* among different groups. Generation time in demographic analyses was inferred based on a synonymous nucleotide mutation rate of 2.94 × 10^−9^ per generation and a 5-year generation interval.

### Phenotyping

A total of 236 *C. pallida* accessions were planted in Danzhou of Hainan Province (109.5E, 19.5N) and Baoshan city of Yunnan Province (98.52E, 24.58 N) during 2021 and 2022. The *C. pallida* plants were planted in August 2021/2022 and harvested in December 2021/2022. Each sample was planted in a single row with seven plants, spaced 0.8 m apart. Phenotyping protocols adhered to the Chinese description specification for tropical grass germplasm resources, covering traits related to plant architecture, stems, leaves, flowers, and seeds. Phenotypic data, including plant height, trifoliolate leaf count, leaf area, developmental timing (days to flowering/seeding), architectural traits (the number of stem branches, inflorescences per plant, flowers per inflorescence, and pods per inflorescence, inflorescence length), and yield components (thousand-seed weight, biomass) were recorded for each samples and average across seven plants.

### Genome-wide association study

GWAS analyses were performed using GEMMA software [19], based on a MLM. This model simultaneously accounts for population stratification and cryptic relatedness. To account for population structure, principal component analysis (PCA) was conducted using genome-wide SNP data, and the top three PCs were included as fixed-effect covariates in the model (Fig. S28). Genetic relatedness among accessions was controlled by incorporating a kinship matrix. The GWAS significant threshold was set at ~1.0 × 10^−5^ to identify trait-associated loci.

## Supplementary Material

Web_Material_uhag026

## Data Availability

All sequencing data of genome and transcriptome are available at the National Genomics Data Center (NGDC, https://ngdc.cncb.ac.cn) database under BioProject number PRJCA009683.
