# Supplementary material for: High-quality genome and variation map reveal valuable loci and the genetic basis of trait divergence driven by geographic dispersal in Crotalaria pallida
Source: Hortic Res. 2026 Jan 29;13(5):uhag026. doi: 10.1093/hr/uhag026 (PMC13148161; doi:10.1093/hr/uhag026)
Supplement: Web_Material_uhag026 [file web_material_uhag026.zip › Supplementary Figures-v2.docx]

Supporting Information

***Crotalaria pallida* genomes reveal valuable loci and the genetic basis of trait divergence driven by geographic dispersal**

Hubiao Yang^1,2#^, Xiaoxue Ye^2,3,4#^, Yiming Wang^5#^, Wei Yan^6^,^#^ Changmian Ji^2,3,4^, Yu Wang^2,3,4^, Zehong Ding^2,3,4^, Weiwei Tie^2,3,4^, Zhenfan Hao^7^, Qian Liu^6^, Zhengyang Zhong^8^, Xuekui Dong^9^, Ling Kang^5^, Mufei Zhu^5^, Hao Lv^5^, Wei Hu^2,3,4^*, Guodao Liu^2^*, Zhibiao Nan^1^*


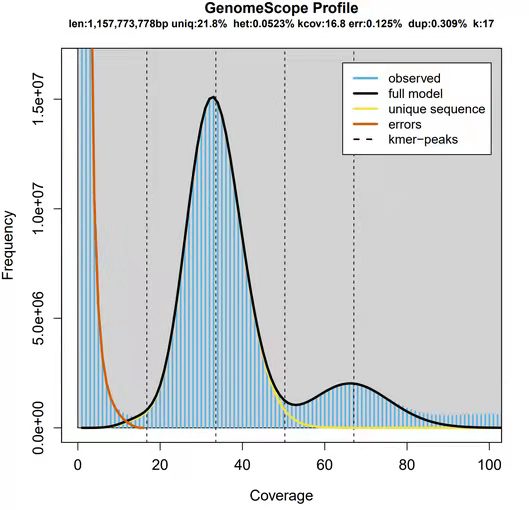


Figure S1. GenomeScope k-mer profile plot for the *C.pallida* genome based on 17-mers in Illumina reads. The observed k-mer frequency distribution is depicted in blue, whereas the GenomeScope fit model is shown as a black line. The unique and putative error k-mer distributions are plotted in yellow and red, respectively.


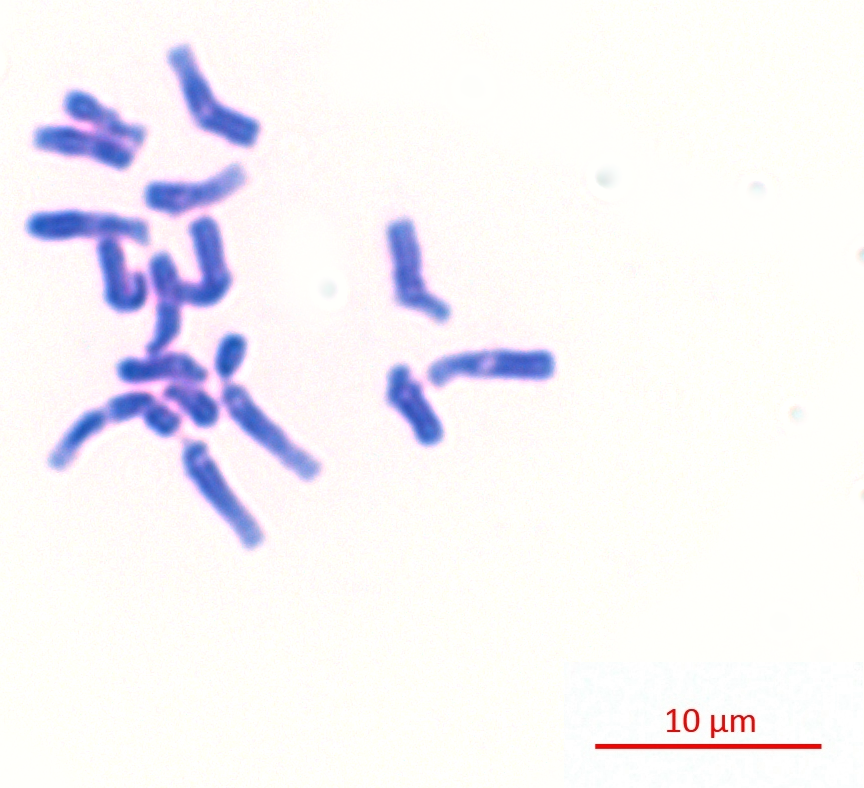


Figure S2. Karyotype analysis showing 8 pairs of chromosomes (2n = 16) in *C. pallida.*


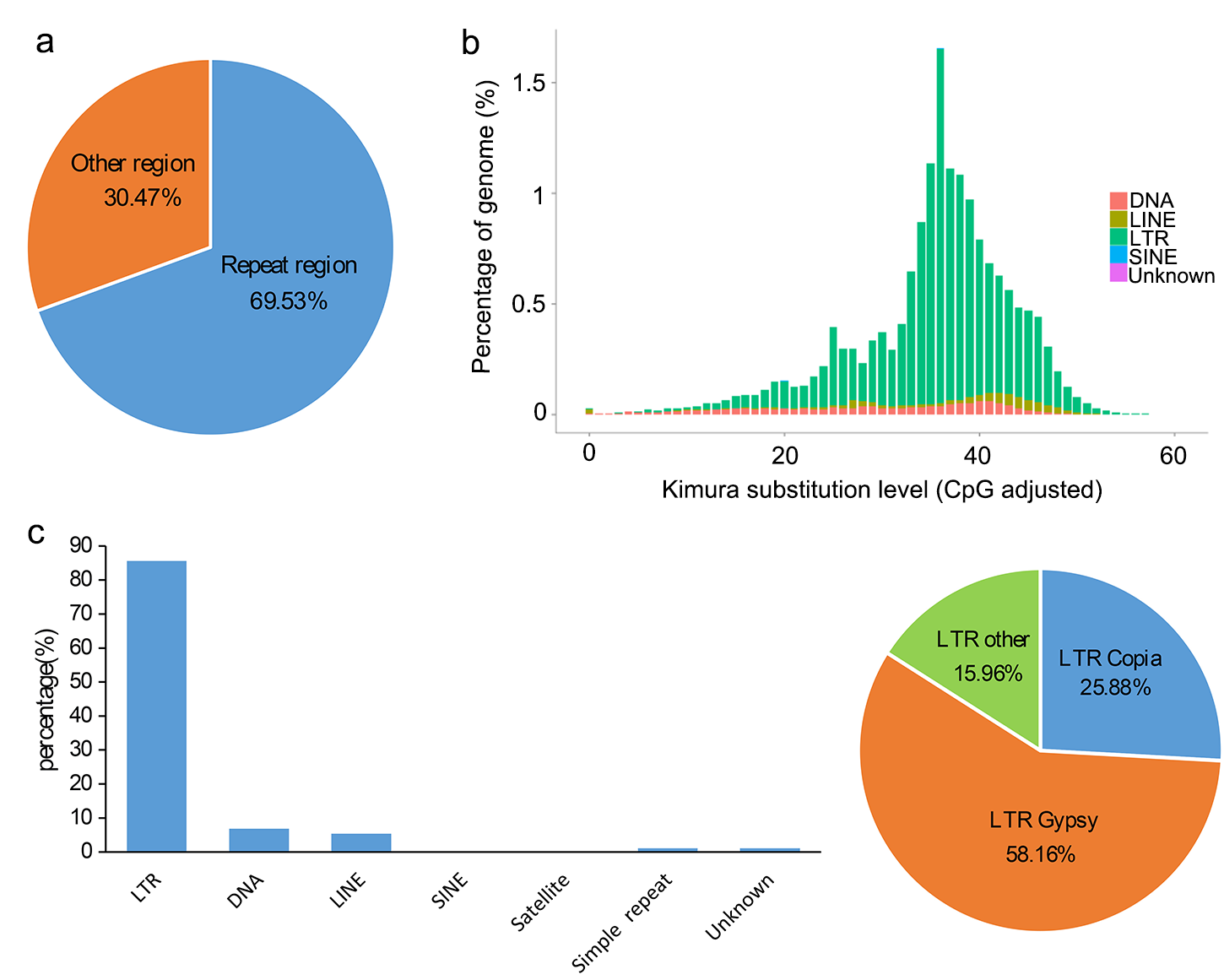


Figure S3. Characteristics of repetitive elements in the *C. pallida* genome. a, The proportion of repetitive elements in the *C. pallida* genome. b, Distribution of divergence degree of TE sequence. c, The proportion of different LTR types.


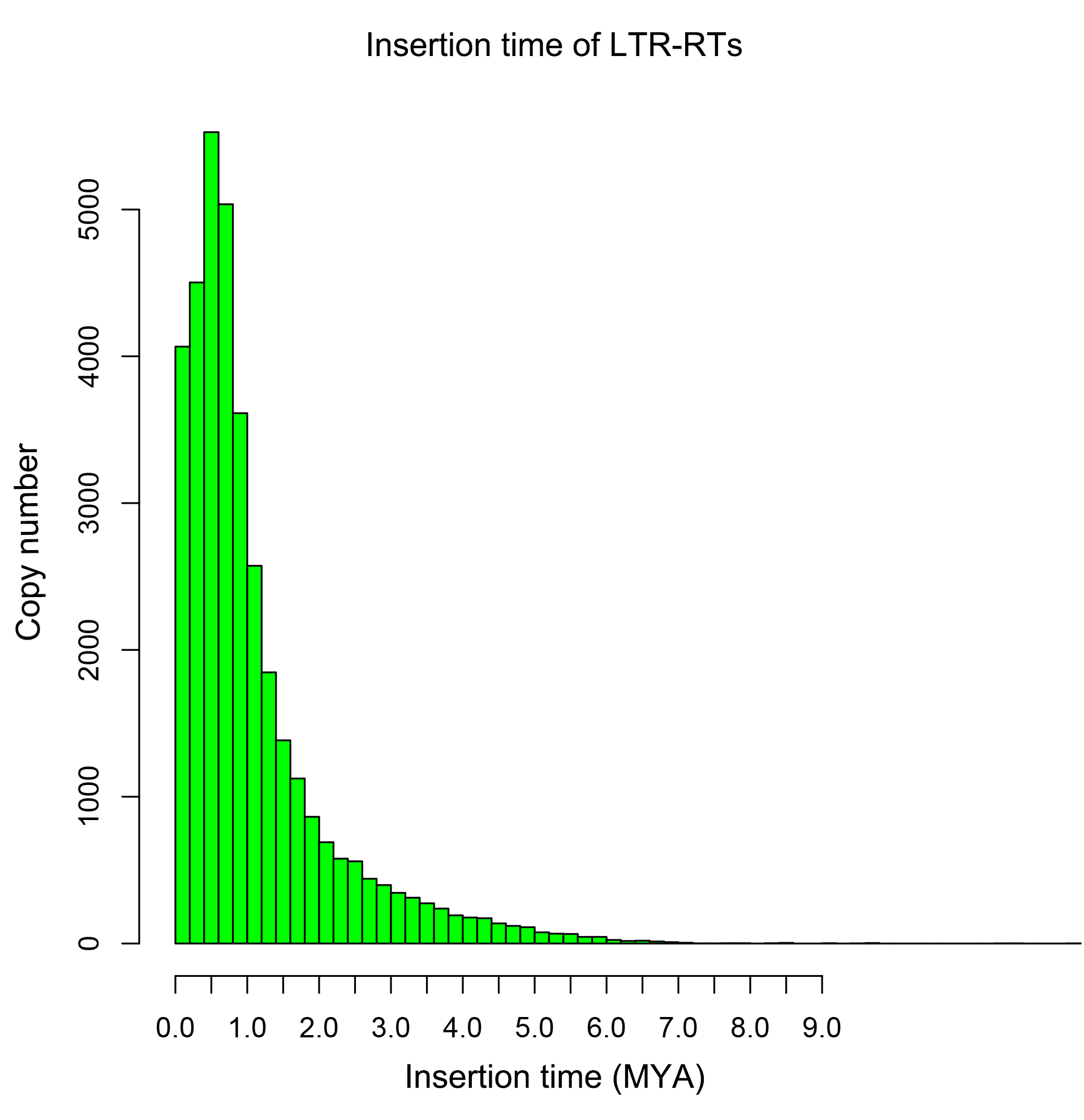


Figure S4. Timing of LTR retrotransposon insertions.


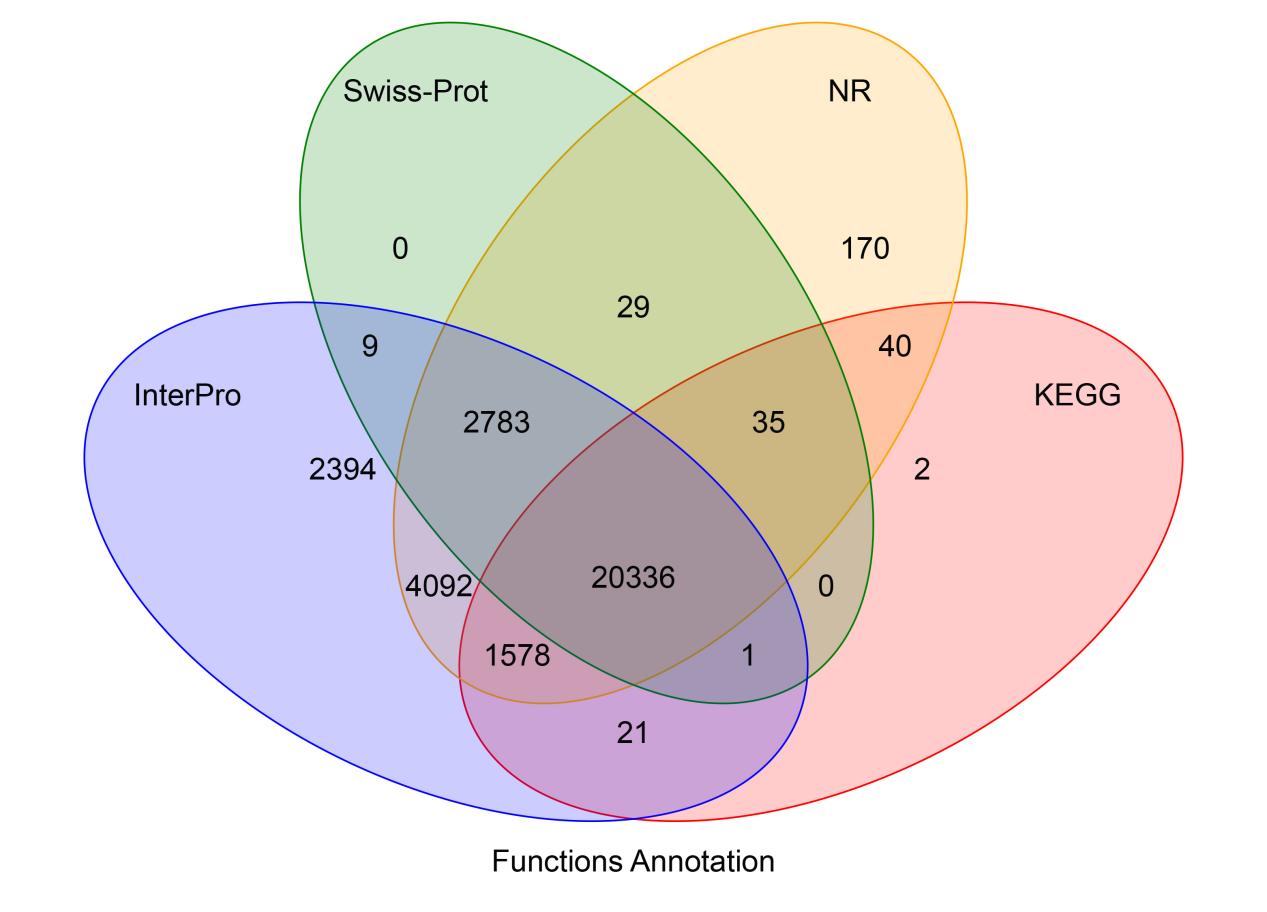


Figure S5. Annotation of gene models using Swiss-Prot, NR, KEGG, and InterPro databases.


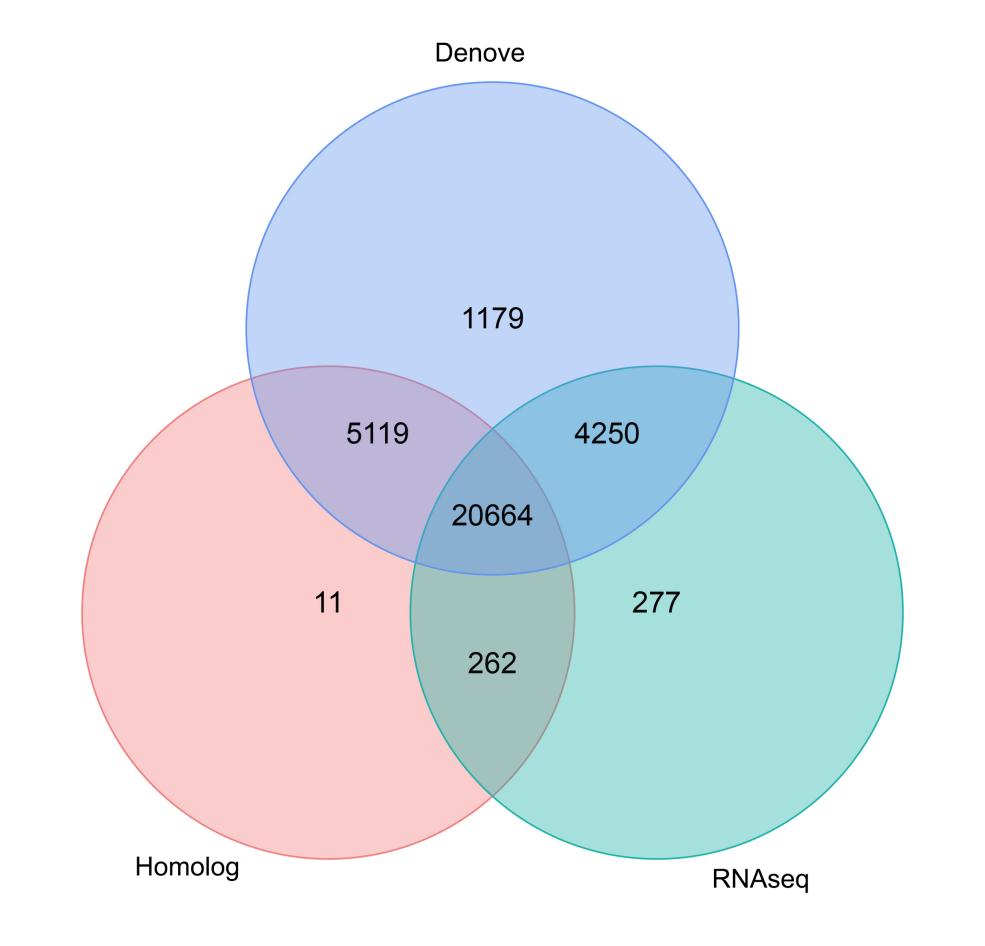


Figure S6. Venn diagram of gene models supported by *de novo*, homology based, and/or transcriptomic evidence.


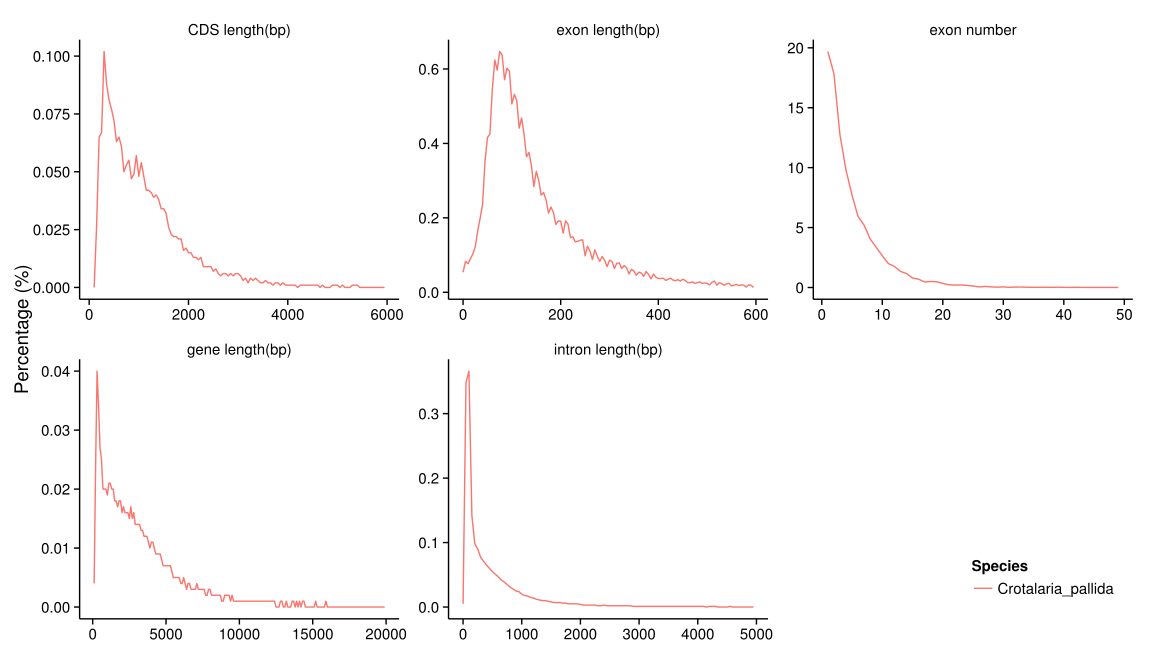


Figure S7. Characteristics of predicted protein-coding genes in *C. pallida.*


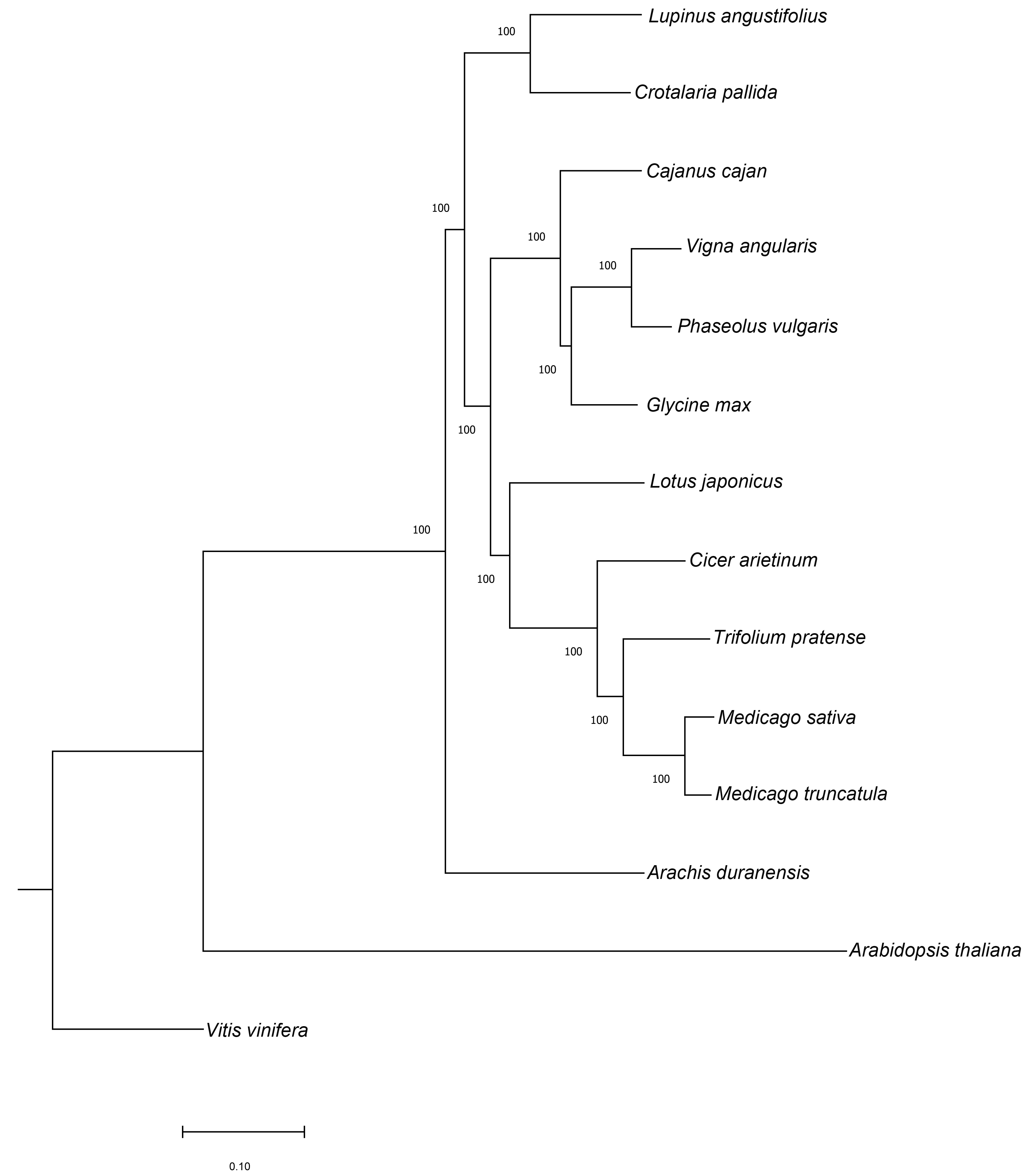


Figure S8. Phylogenetic tree on the basis of single-copy orthologous genes shared among *C. pallida* and 13 other plant species.


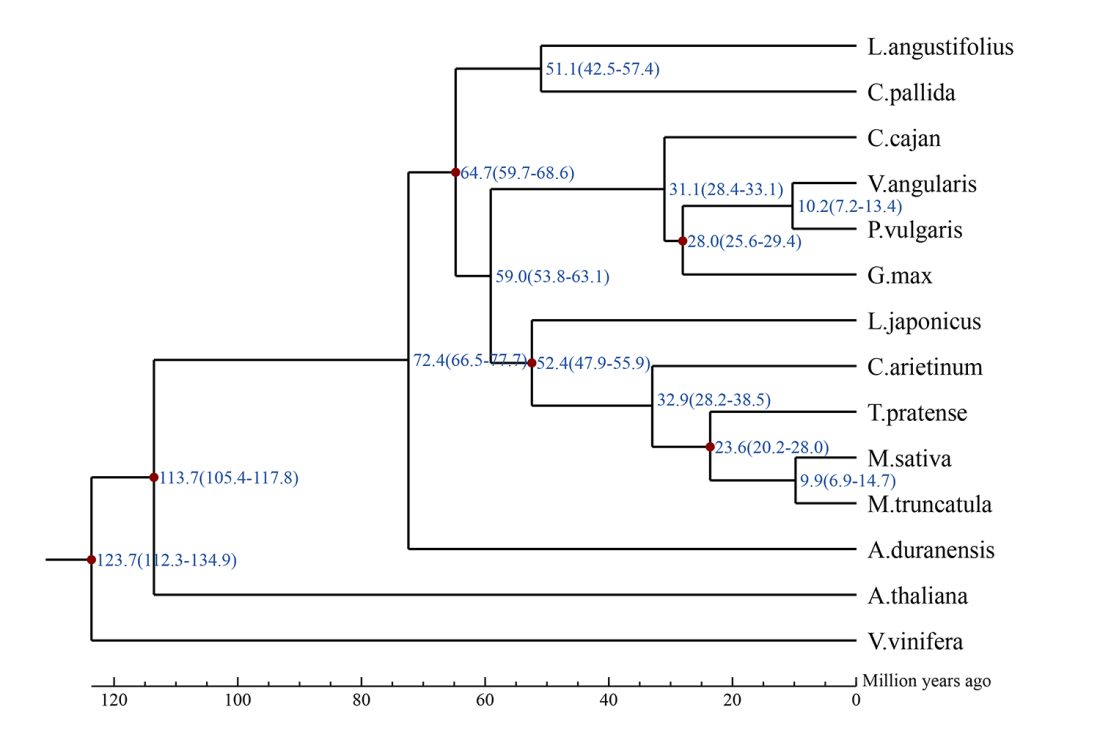


Figure S9. Estimation of the divergence time of the *C. pallida* with 13 other species based on orthologous relationships. Blue numbers at the nodes are divergence time to present (MYA).


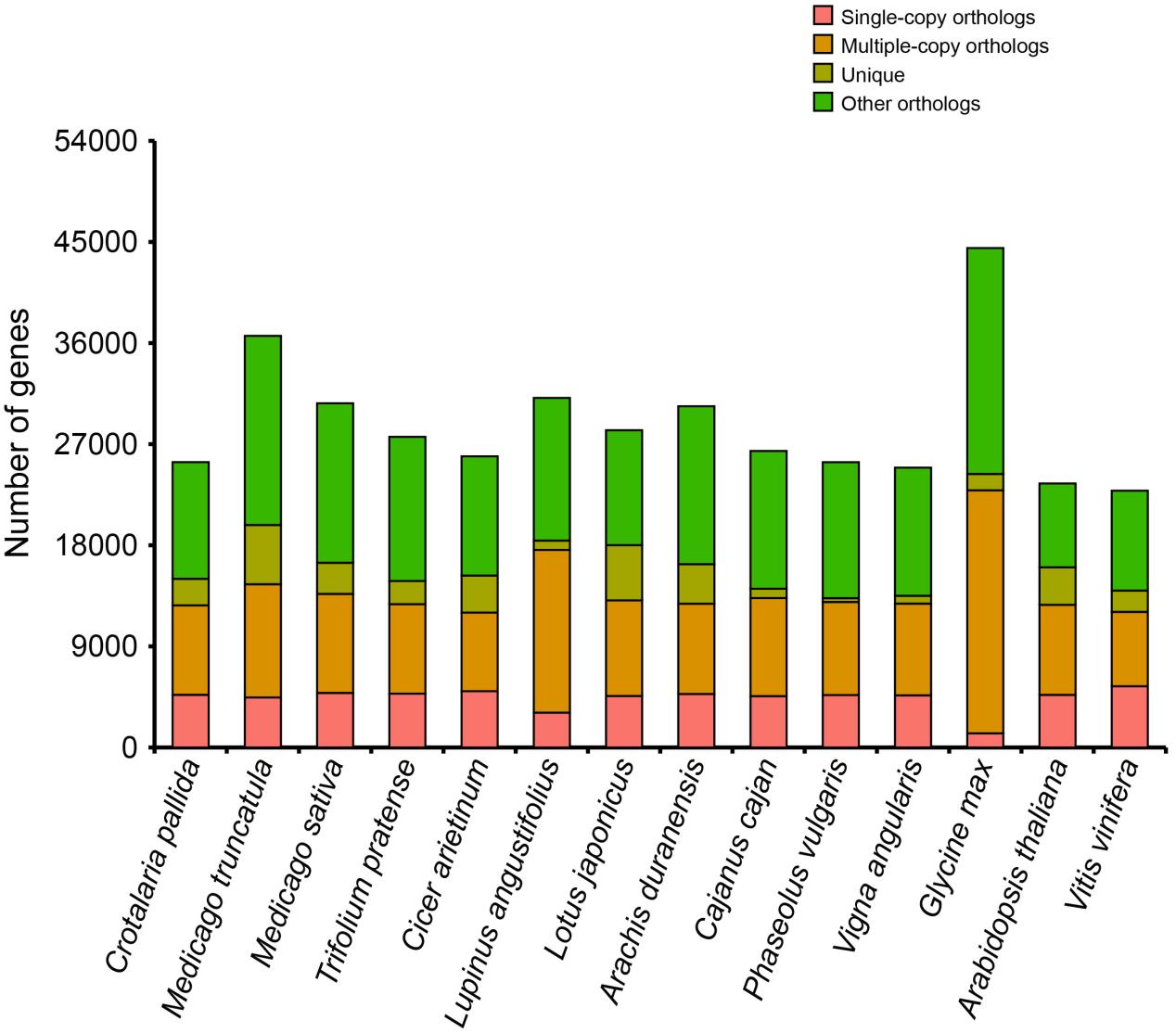


Figure S10. Gene numbers in each category that were defined by OrthoMCL.

*
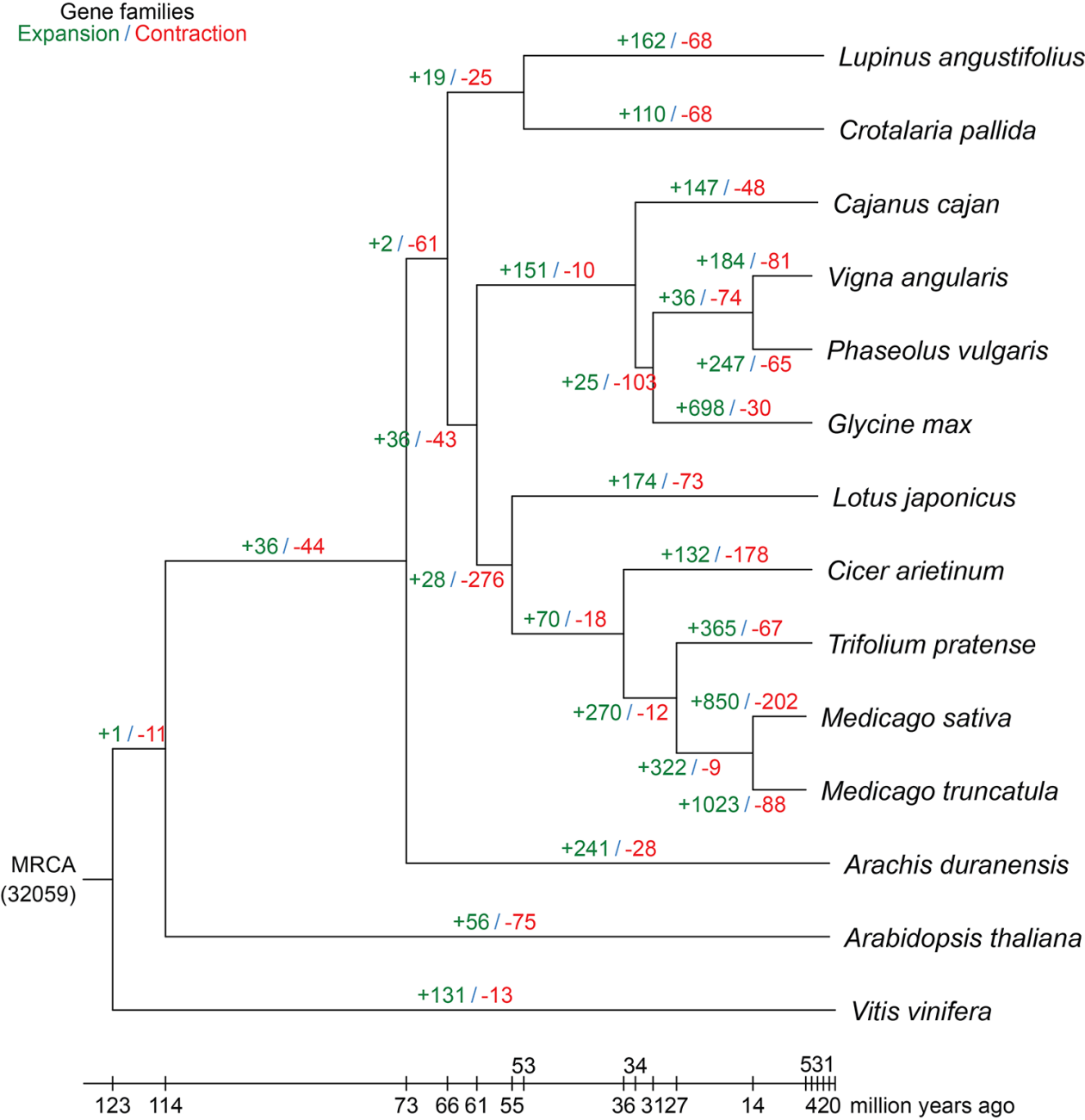
*

Figure S11. Phylogenetic relationship and the expansion and contraction of gene families. Gene family expansions are indicated in green, and gene family contractions are indicated in red.


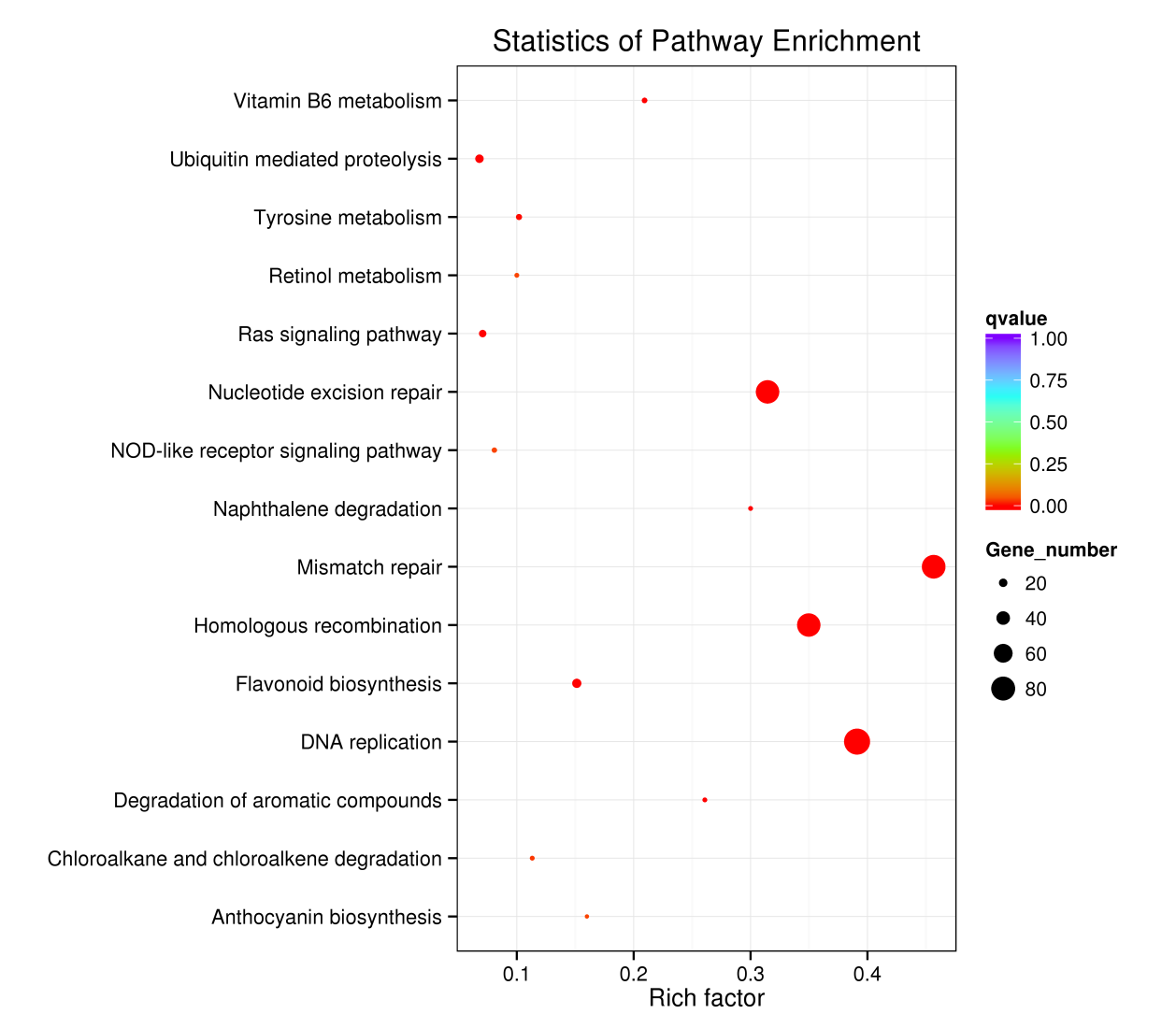


Figure S12. KEGG enrichment of the expanded genes in *C. pallida.*


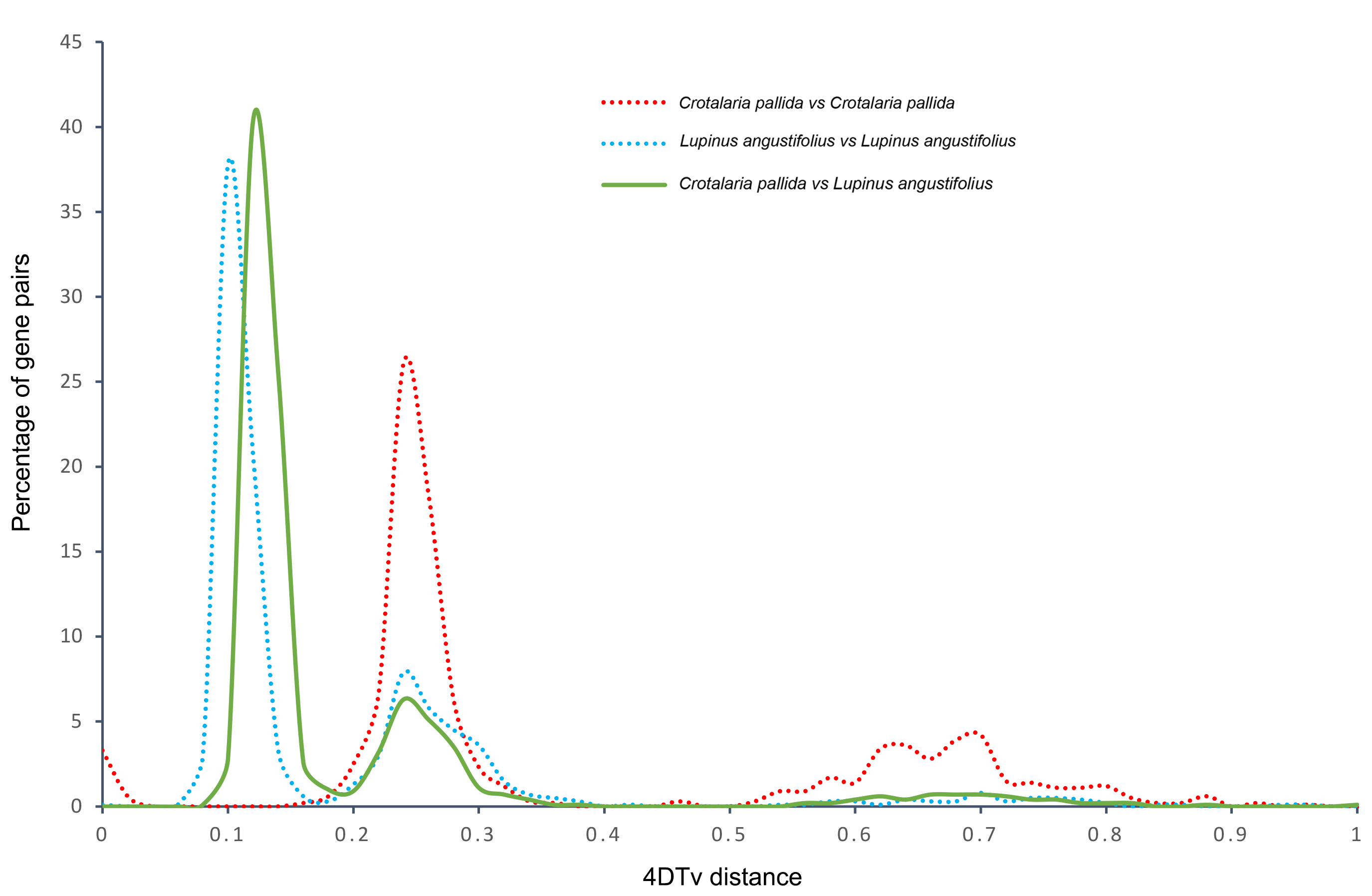


Figure S13. Distribution of the 4DTv distance between duplicated genes in syntenic blocks between genomes of *C. pallida* and *L. angustifolius.*

*
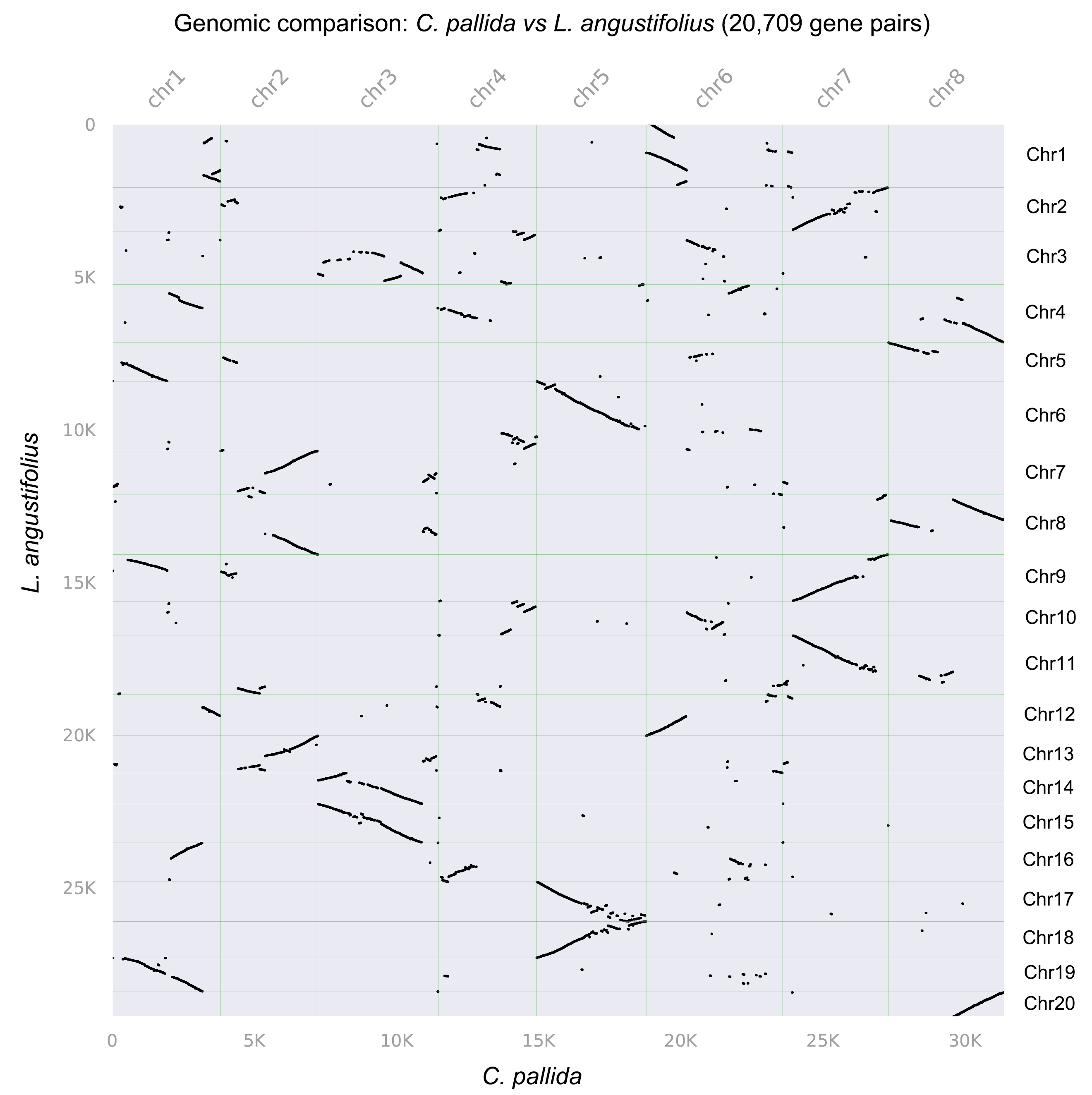
*

Figure S14. Dot plotting of syntenic genes between genomes of *C. pallida* and *L. angustifolius.*

*
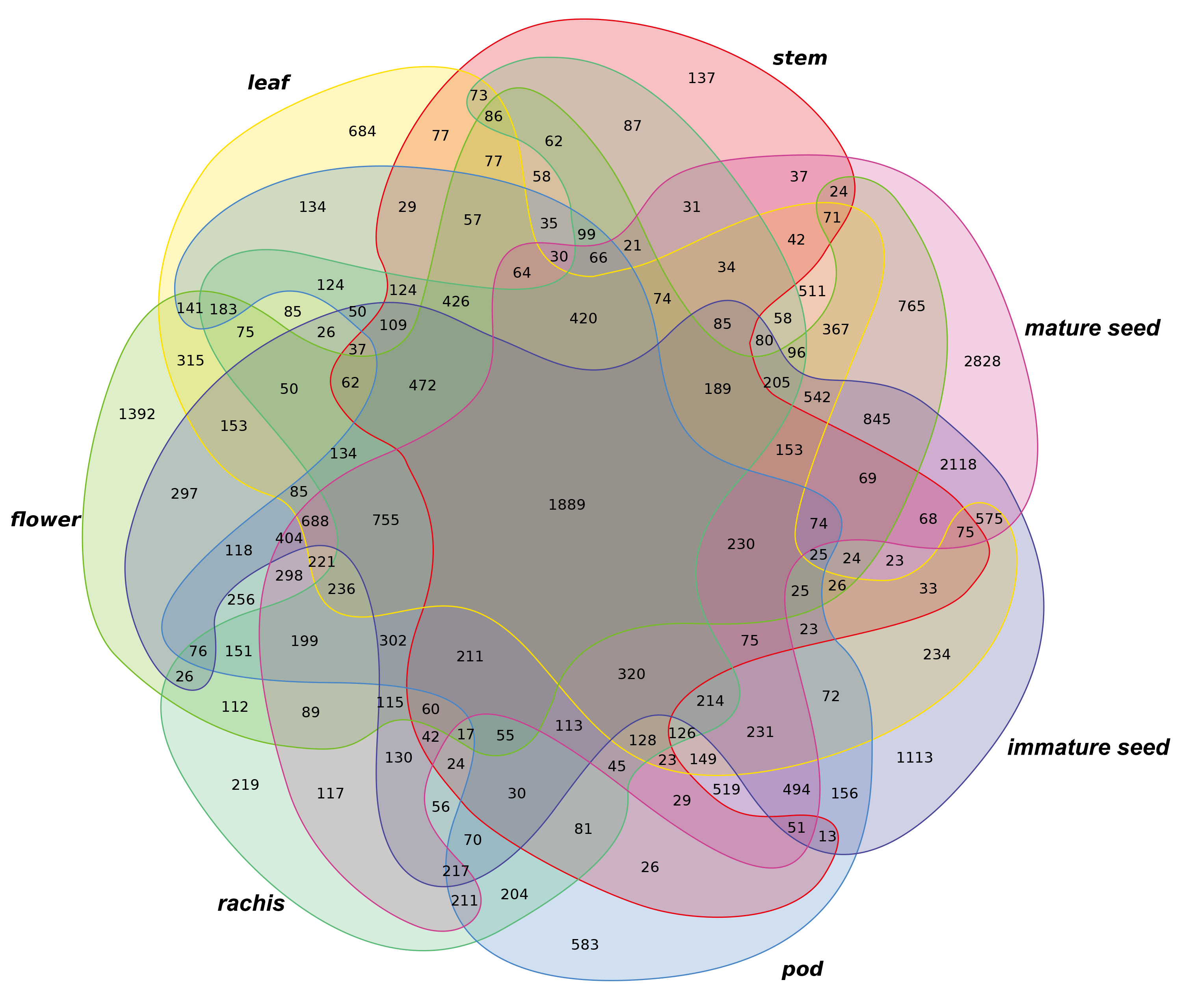
*

Figure S15. Venn diagram of differentially expressed genes in different tissues compared to root.


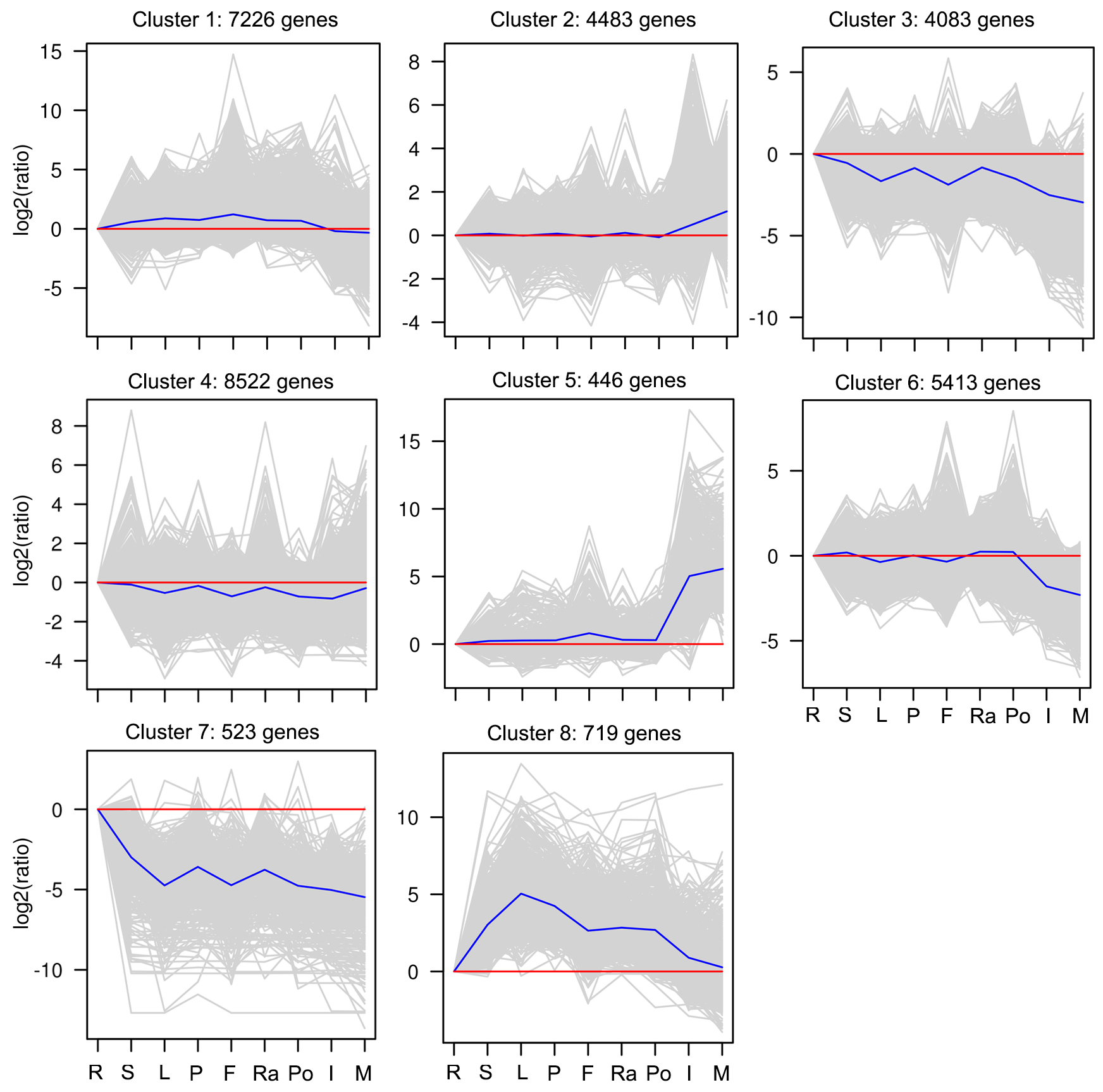


Figure S16. Expression profiles of DEGs in stem (S), leaf (L), petiole (P), flower (F), rachis (Ra), pod (Po), immature seed (I), and mature seed (M) relative to root (R).


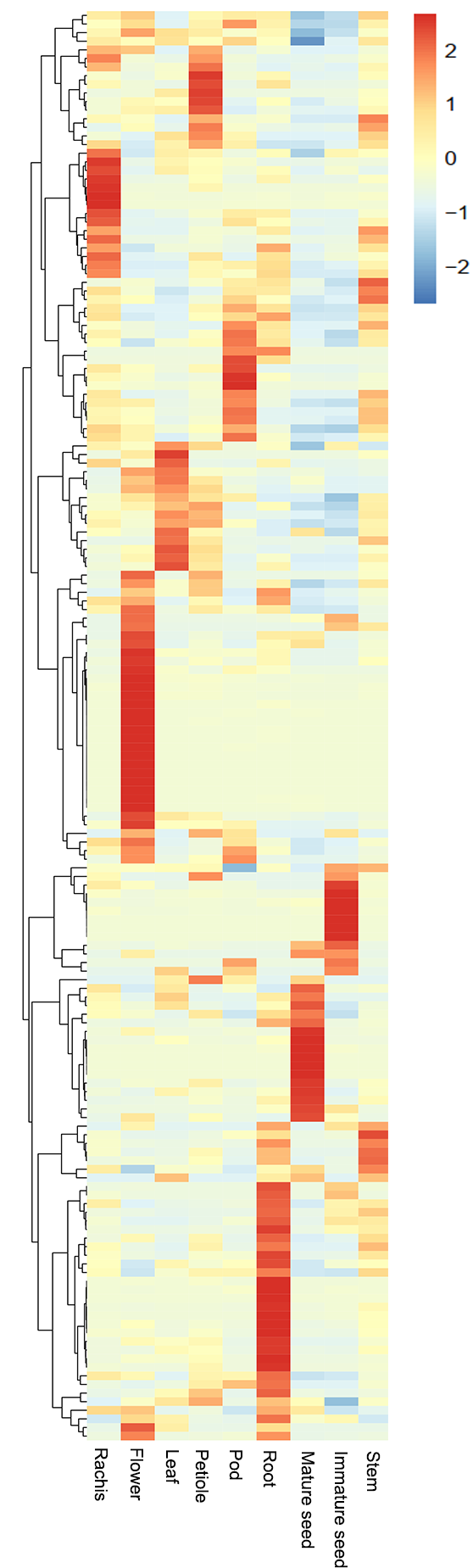


Figure S17. Expression patterns of the genes involved in flavonoid biosynthesis in different tissues.


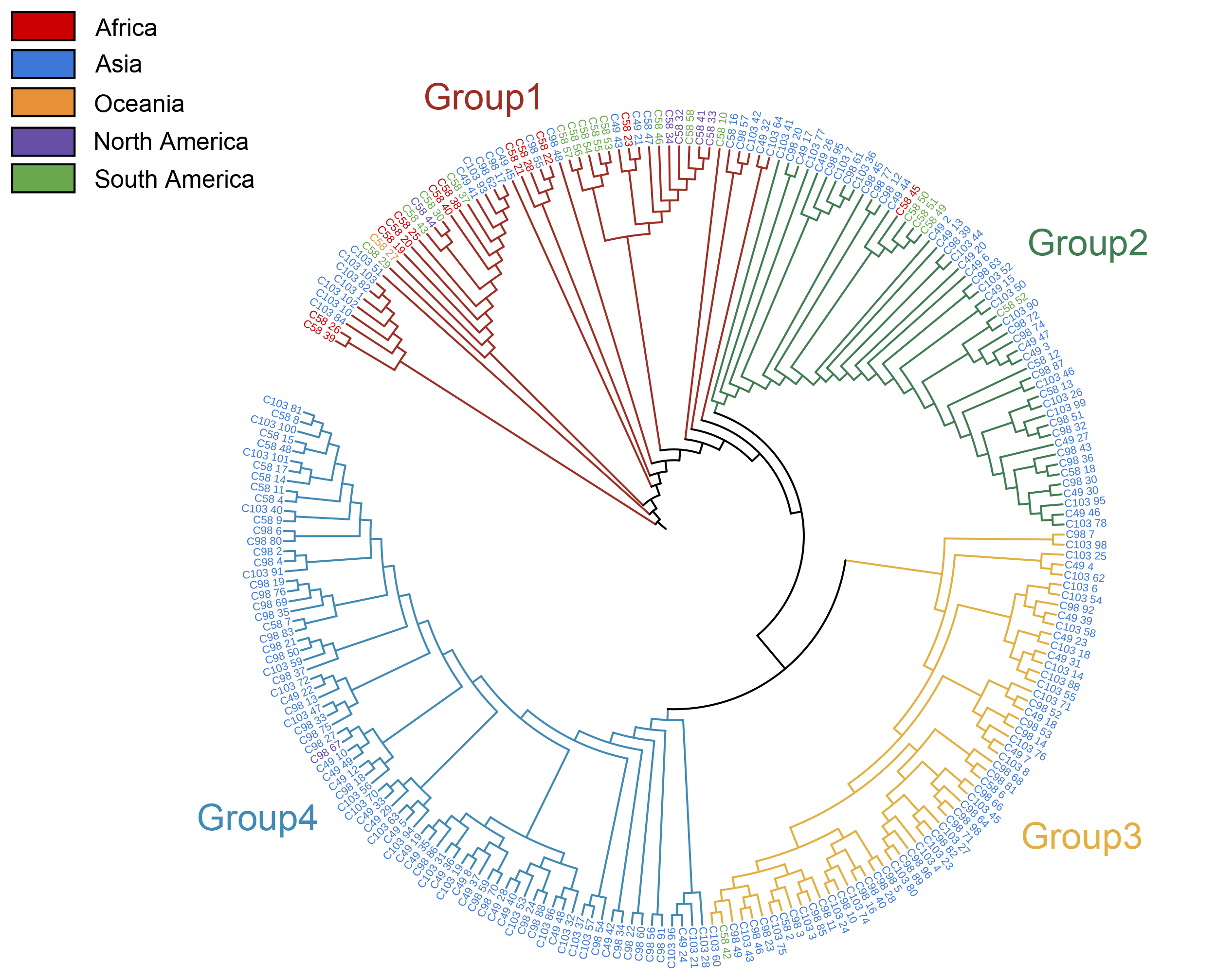


Figure S18. Phylogeny of 236 *C. pallida* accessions generated using the neighbor-joining tree method with genome-wide SNPs.


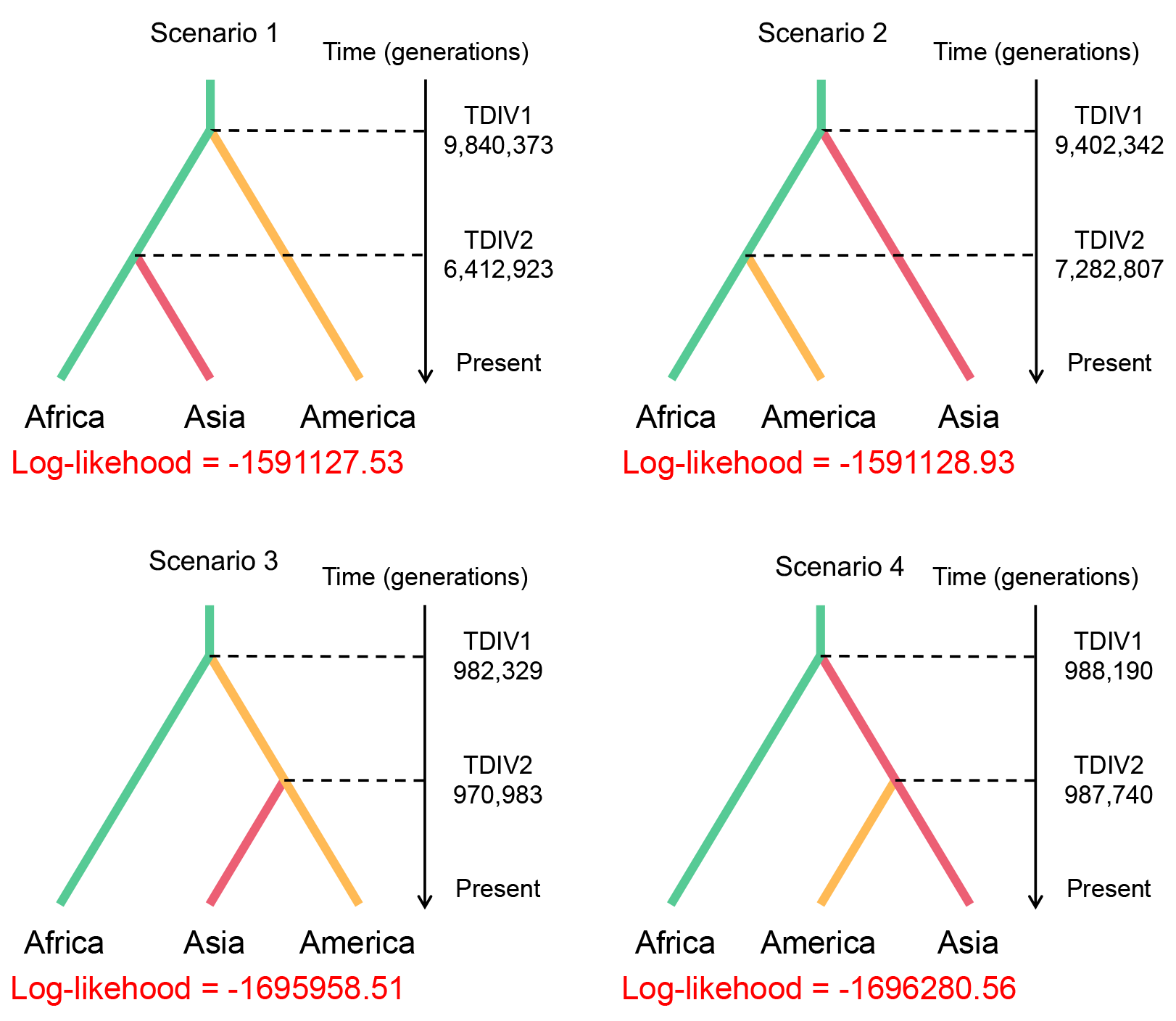


Figure S19. The demographic history of the three groups (Africa, America, and Asia) of *C. pallida*. The Log-likelihood value is indicated under each model. The divergence time for different split events was estimated using fastSimcoal3 and marked on the right of the corresponding event.


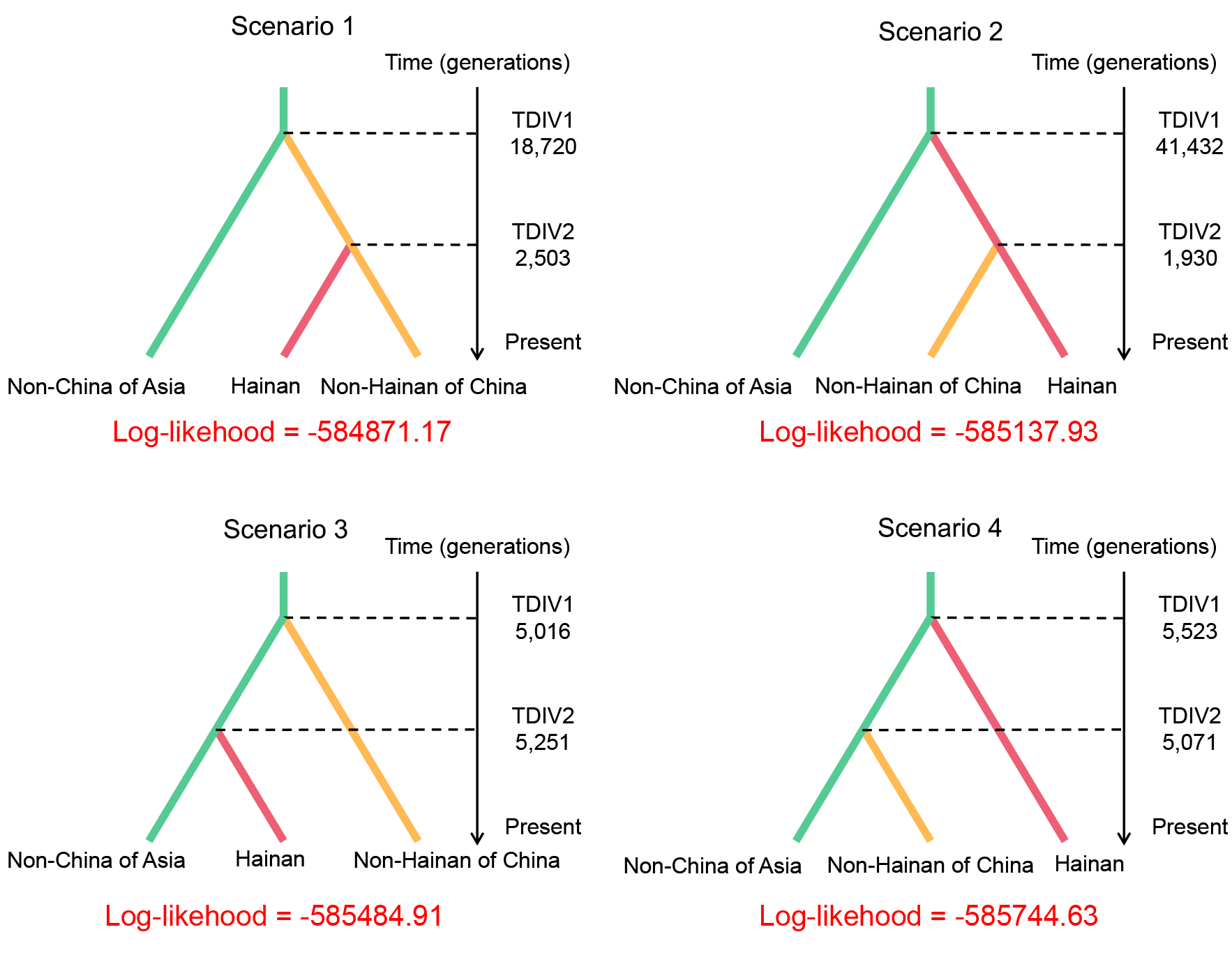


Figure S20. The demographic history of the three groups (Non-China of Asia, Non-Hainan of China, and Hainan) of *C. pallida*. The Log-likelihood value is indicated under each model. The divergence time for different split events was estimated using fastSimcoal3 and marked on the right of the corresponding event.


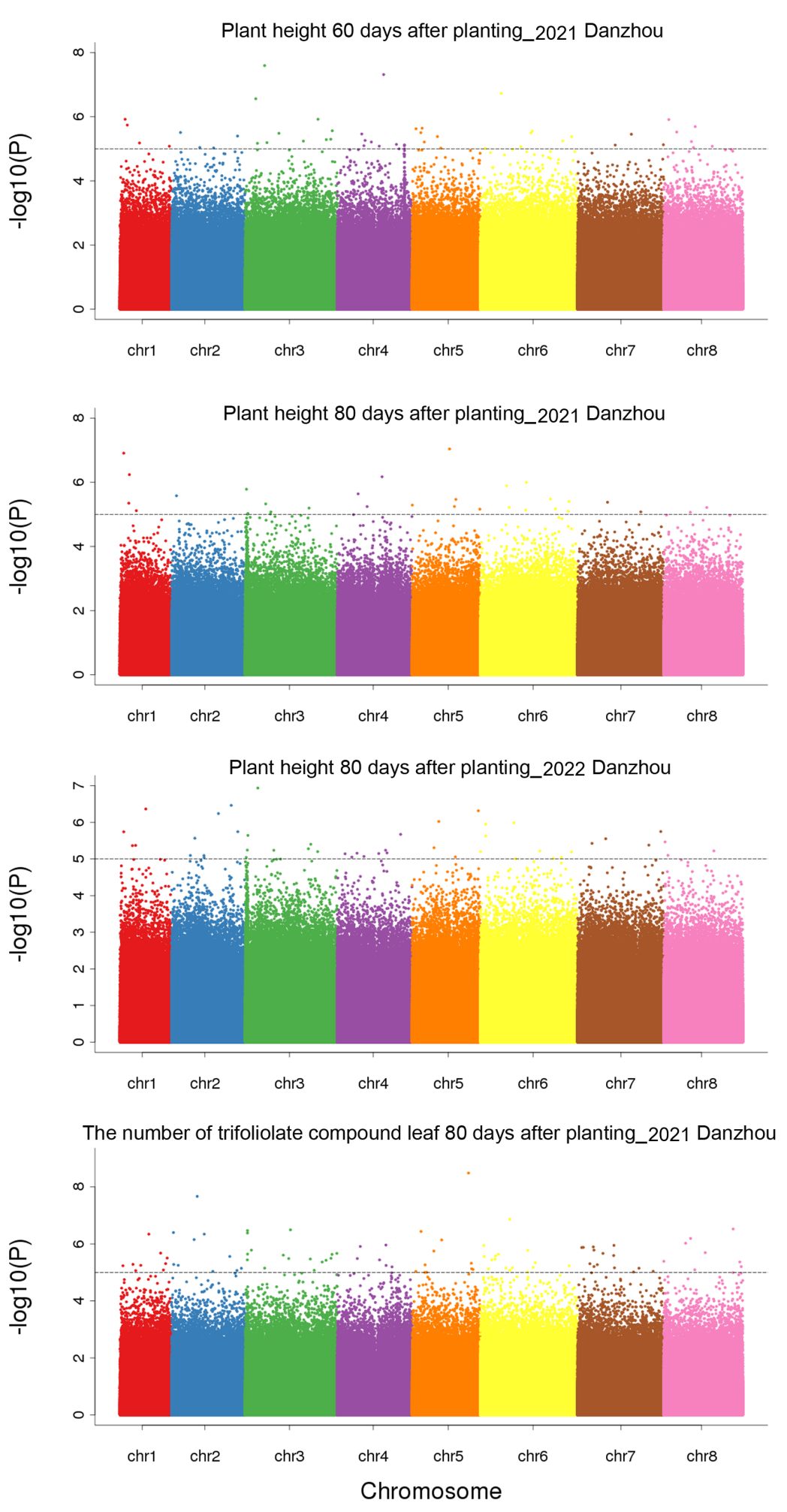


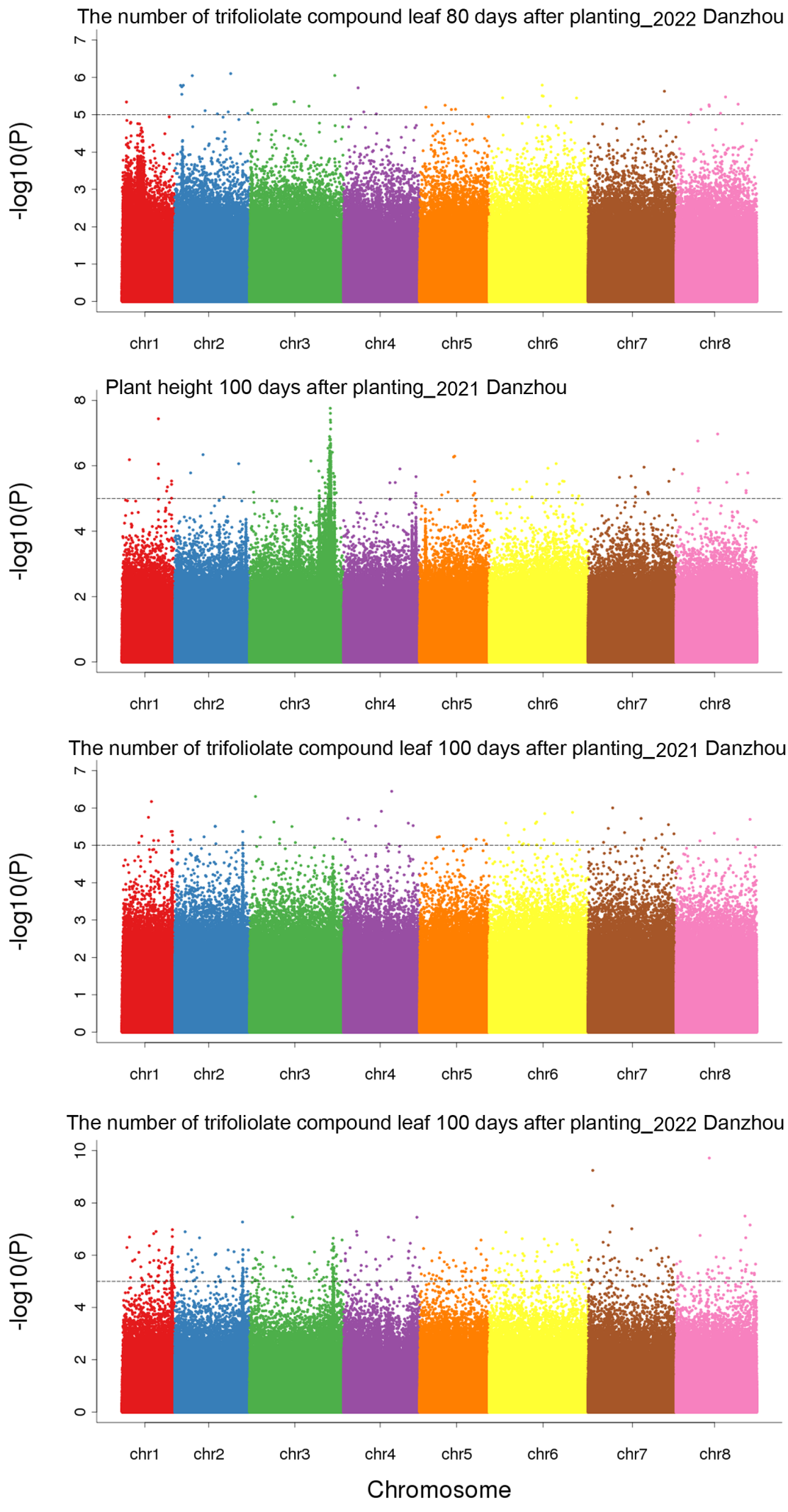


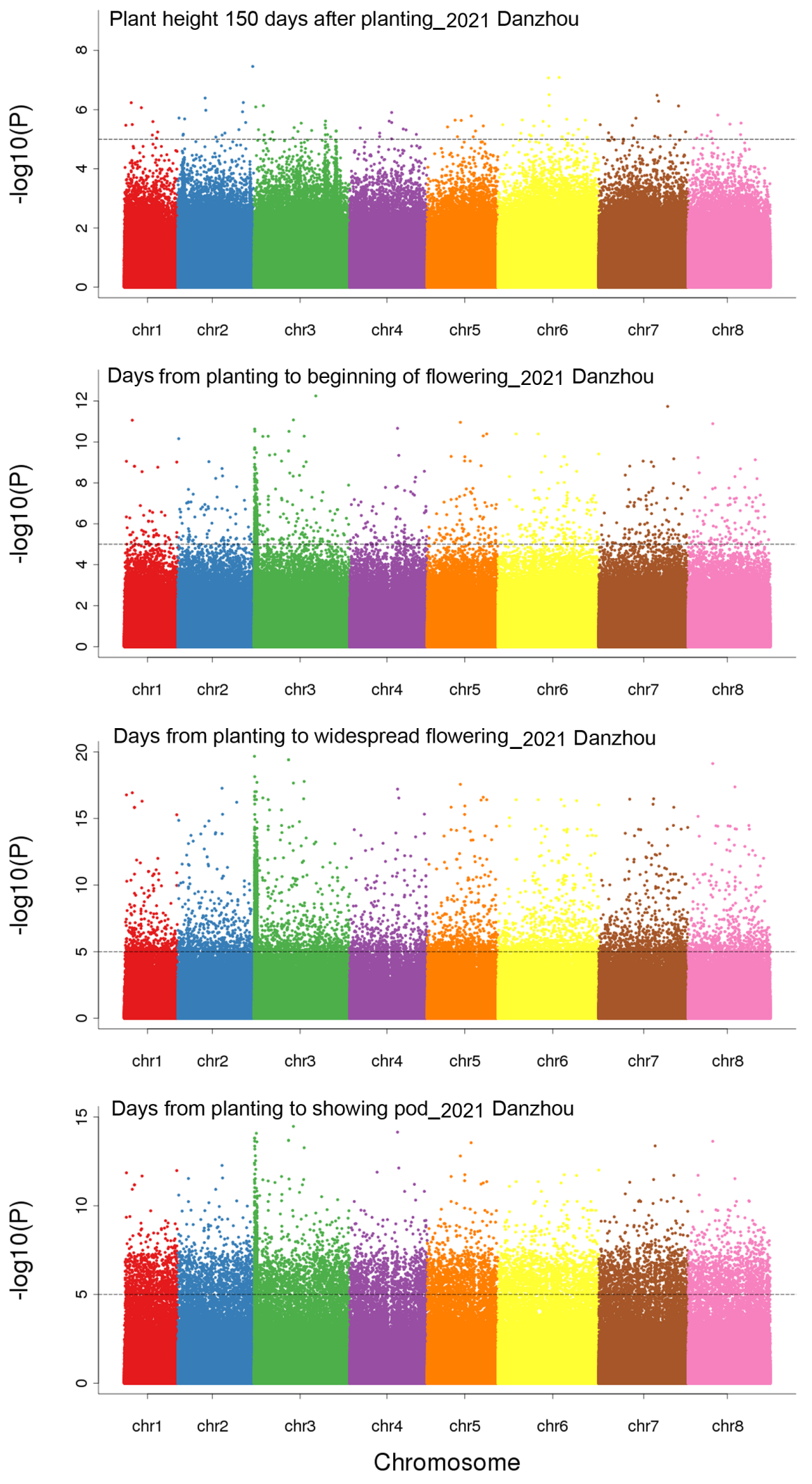


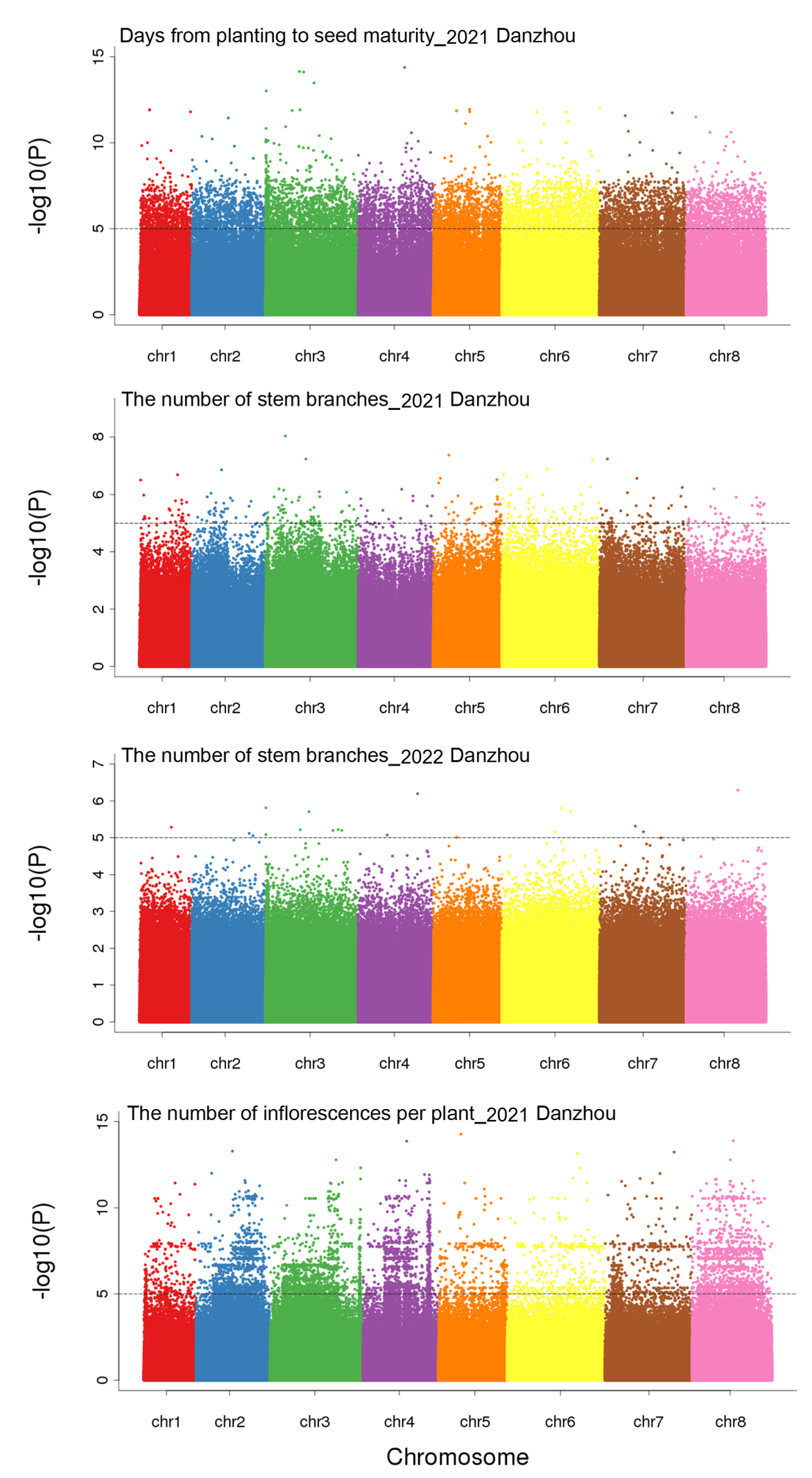


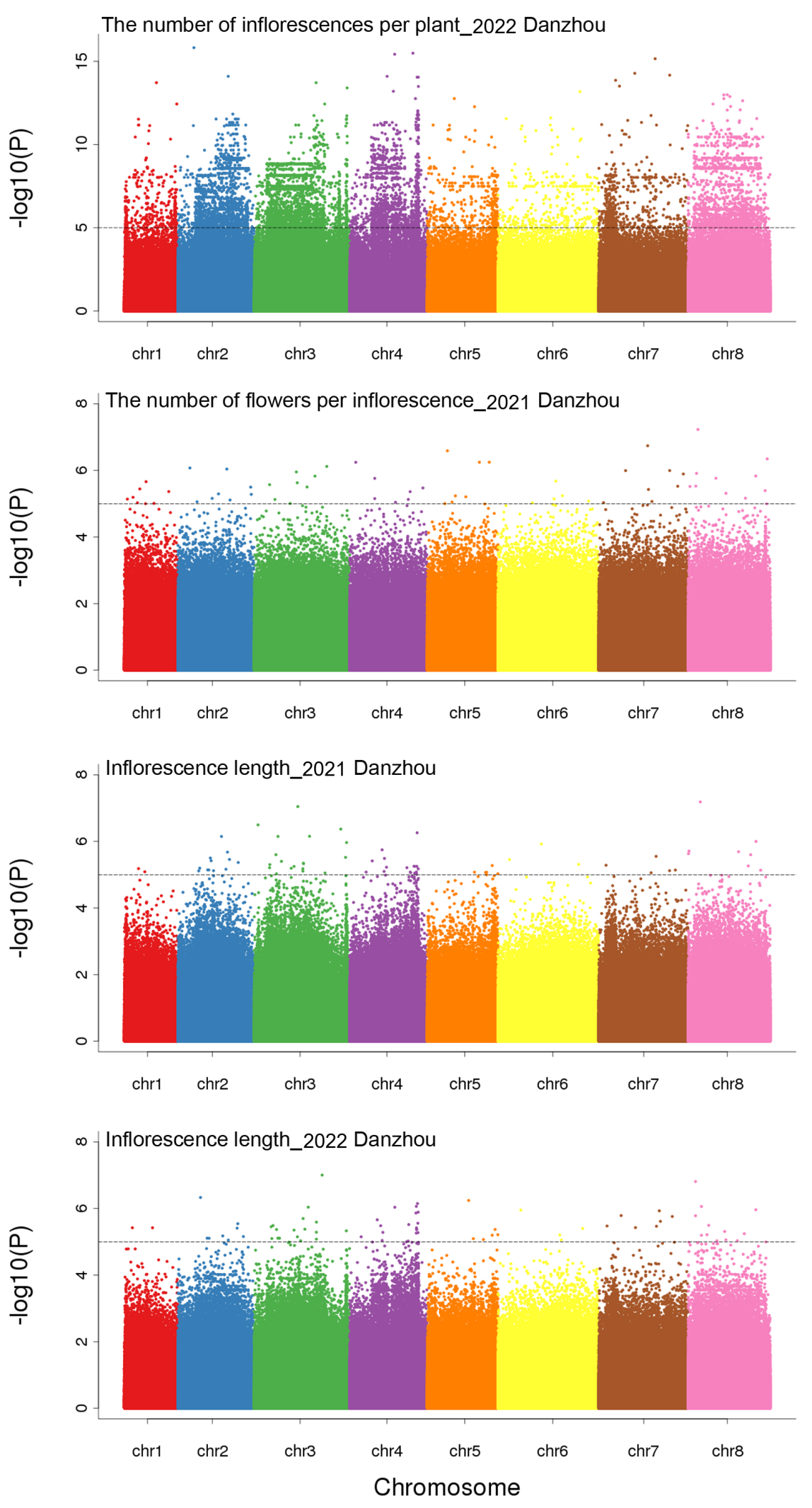


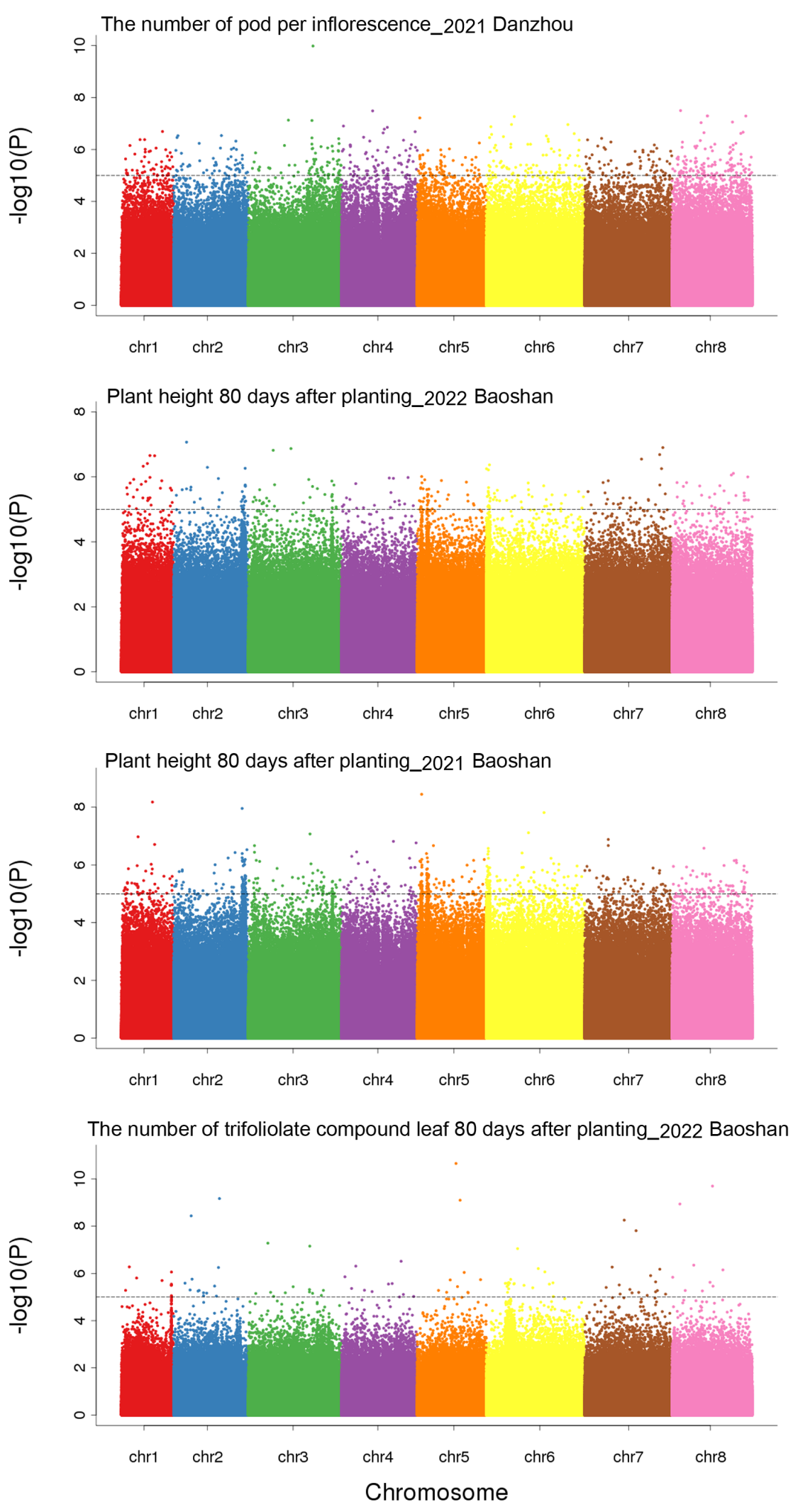


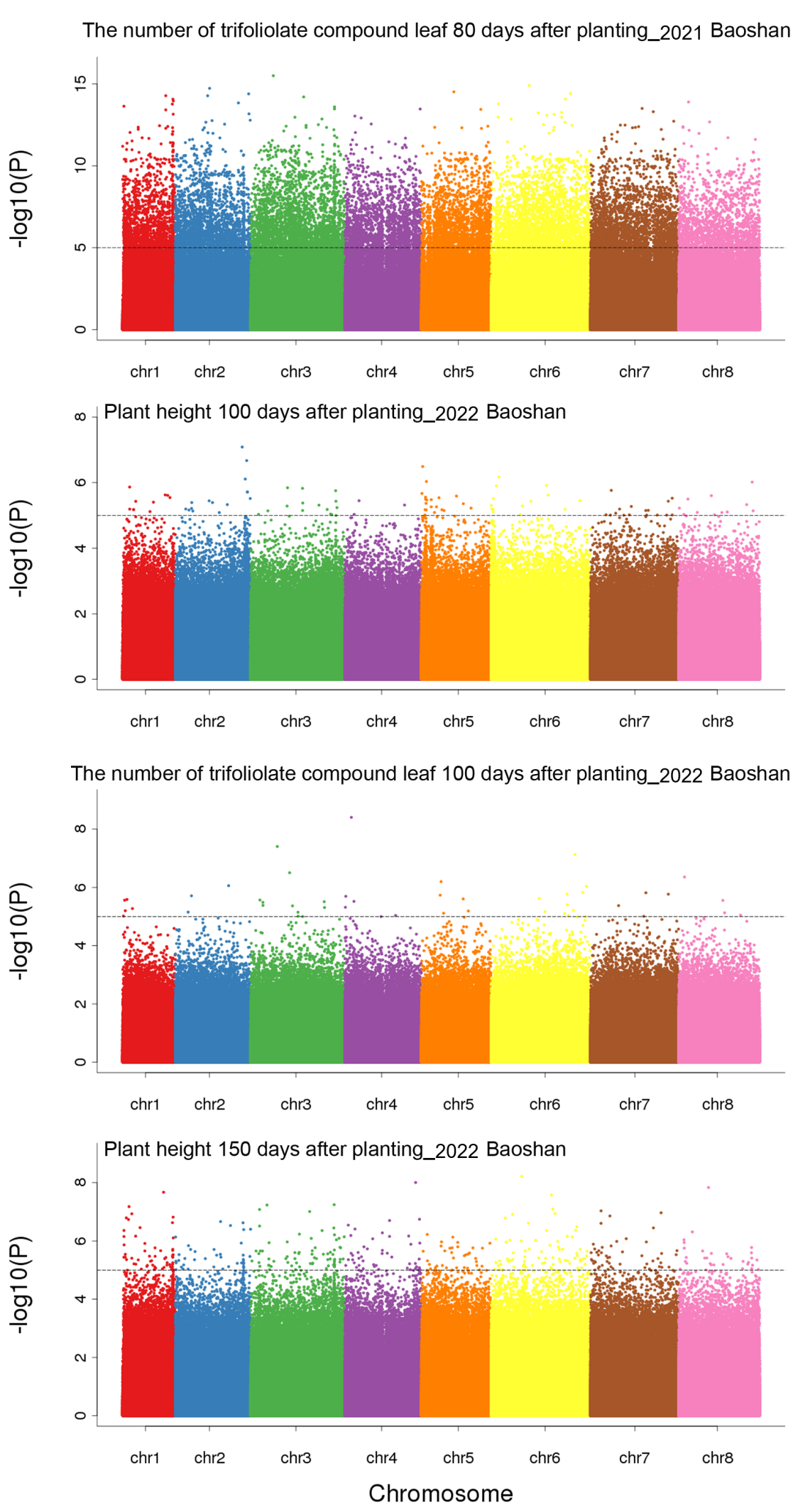


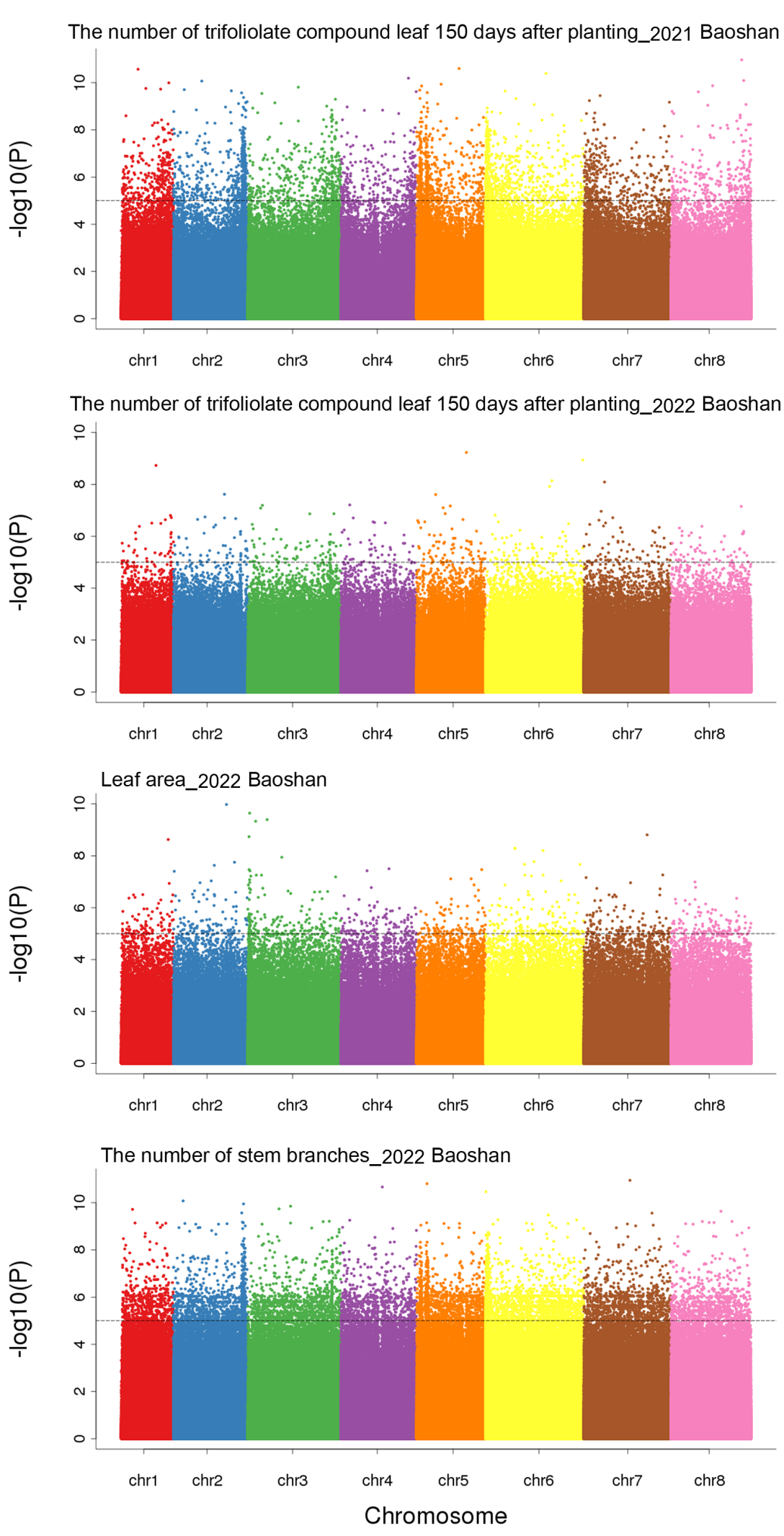


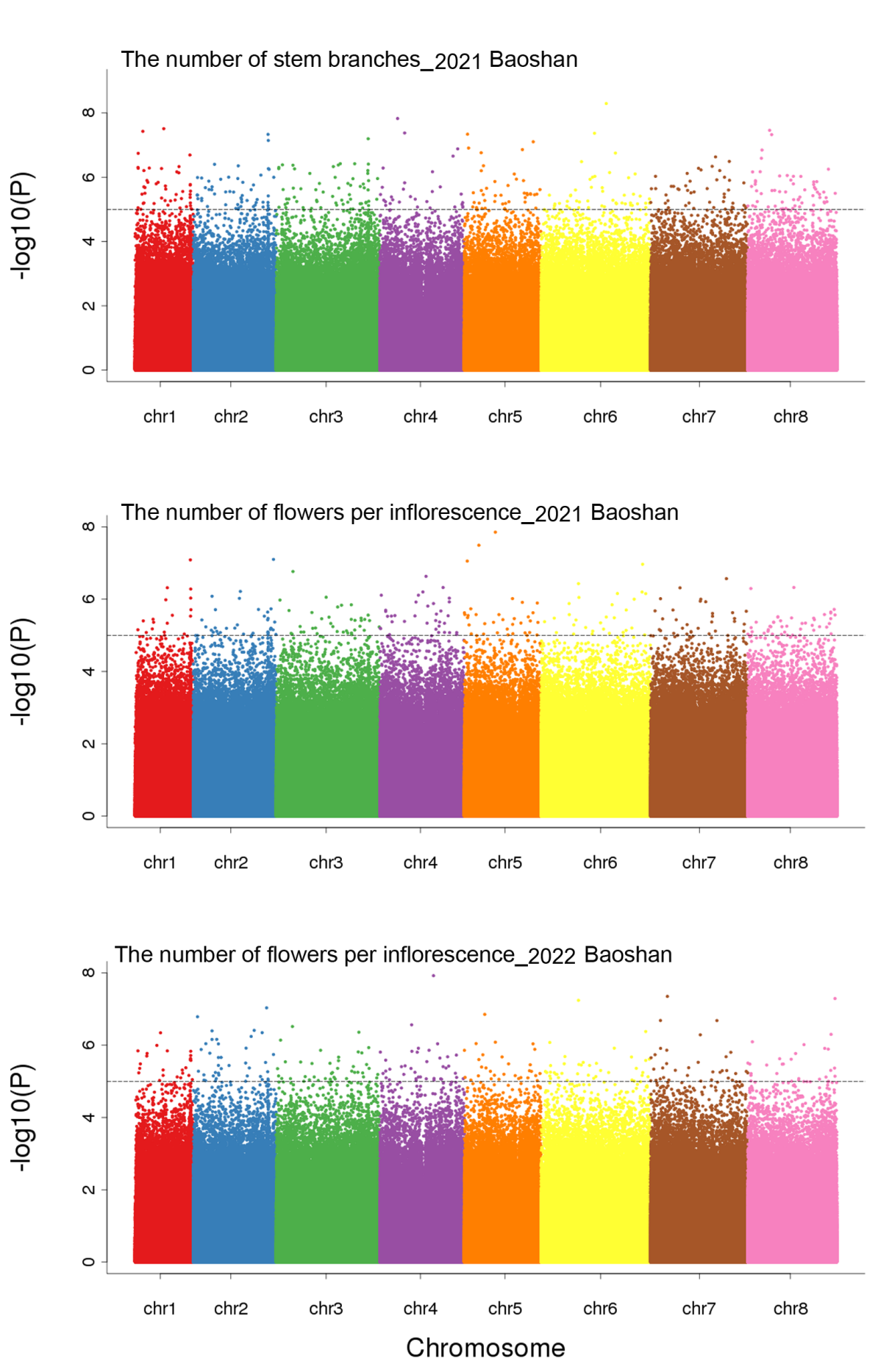


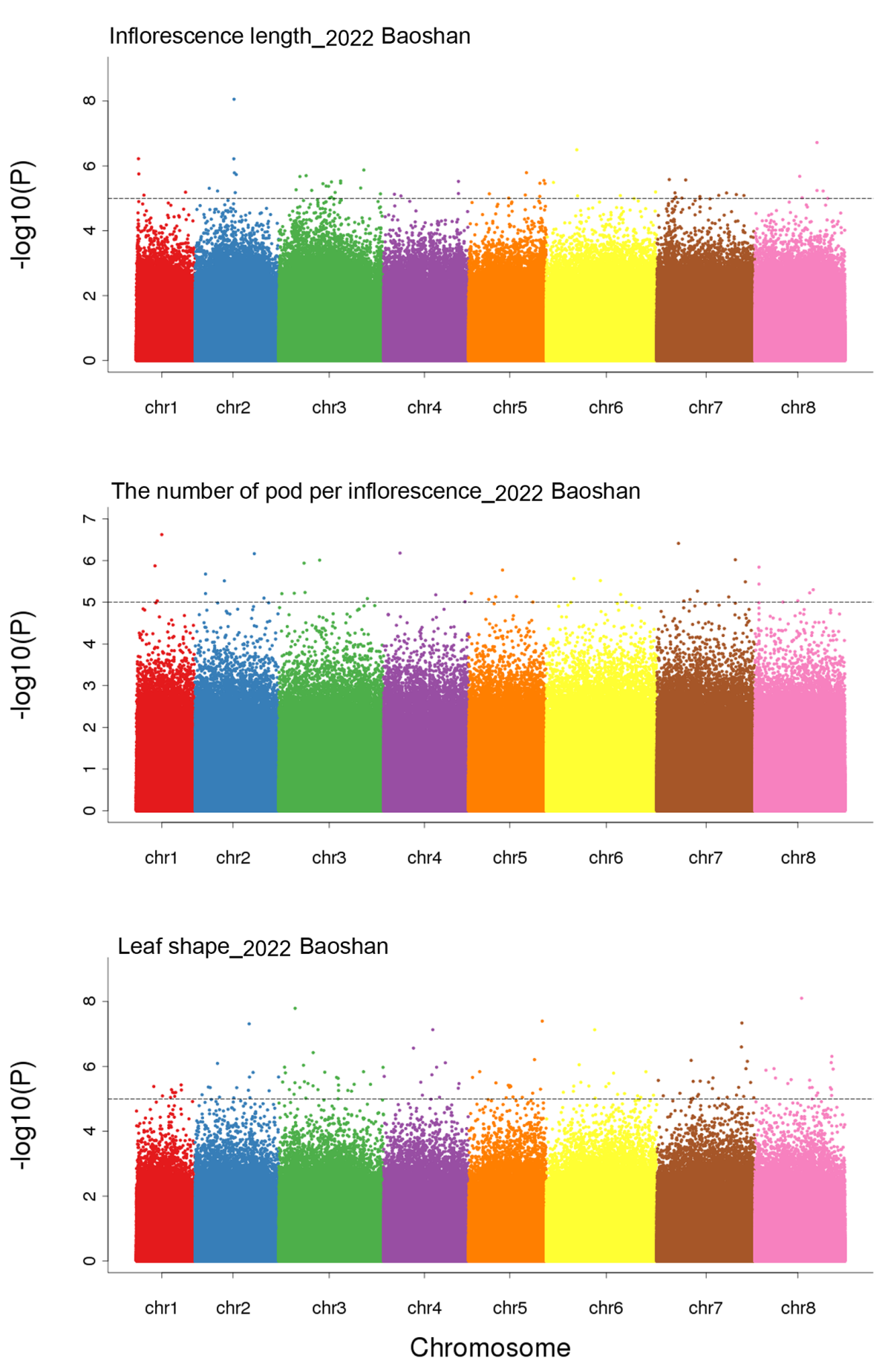


Figure S21. Manhattan plots for GWAS analysis of *C. pallida* agronomic traits.


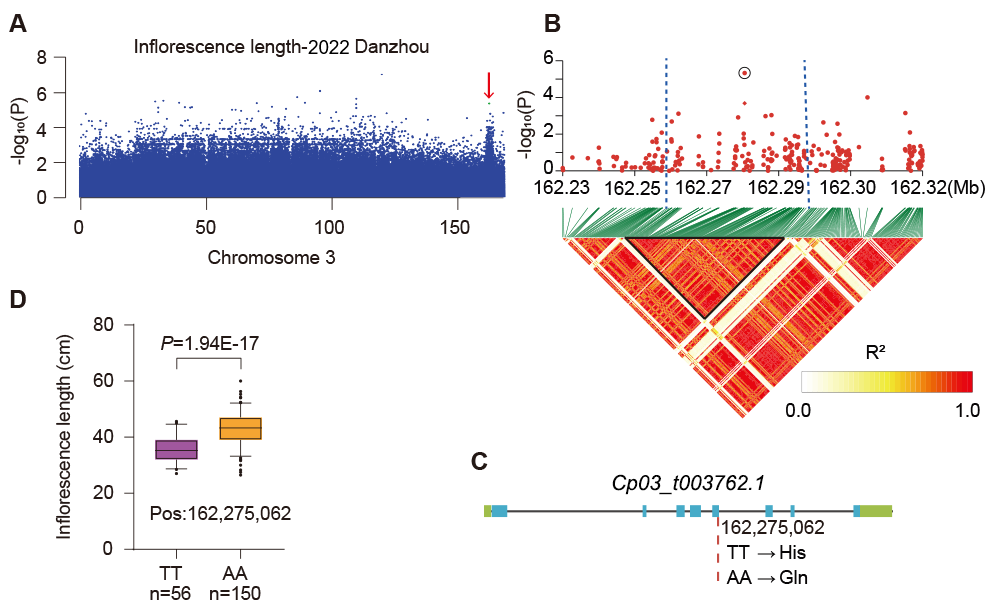


Figure S22. GWAS identification of *evm.TU.contig20.235* as a candidate gene for inflorescence length on chromosome 3. (A) Manhattan plots for inflorescence length on chromosome 3 using GEMMA-MLM. Red arrow indicates the significant GWAS peak. (B) Local manhattan plot (top) and linkage disequilibrium heat map (bottom) surrounding the GWAS signal. Blue dashed lines represent the candidate region. The core SNP in the candidate gene is circled. (C) *Cp03_t003762.1* gene model. The dashed red line indicates the position of nonsynonymous SNP. (D) Comparison of inflorescence length based on the haplotypes in the candidate gene. The center line represents the median, box limits indicate the upper and lower quartiles, and whiskers denote the range of the data. The significance of difference was derived with two-tailed *t*-test.


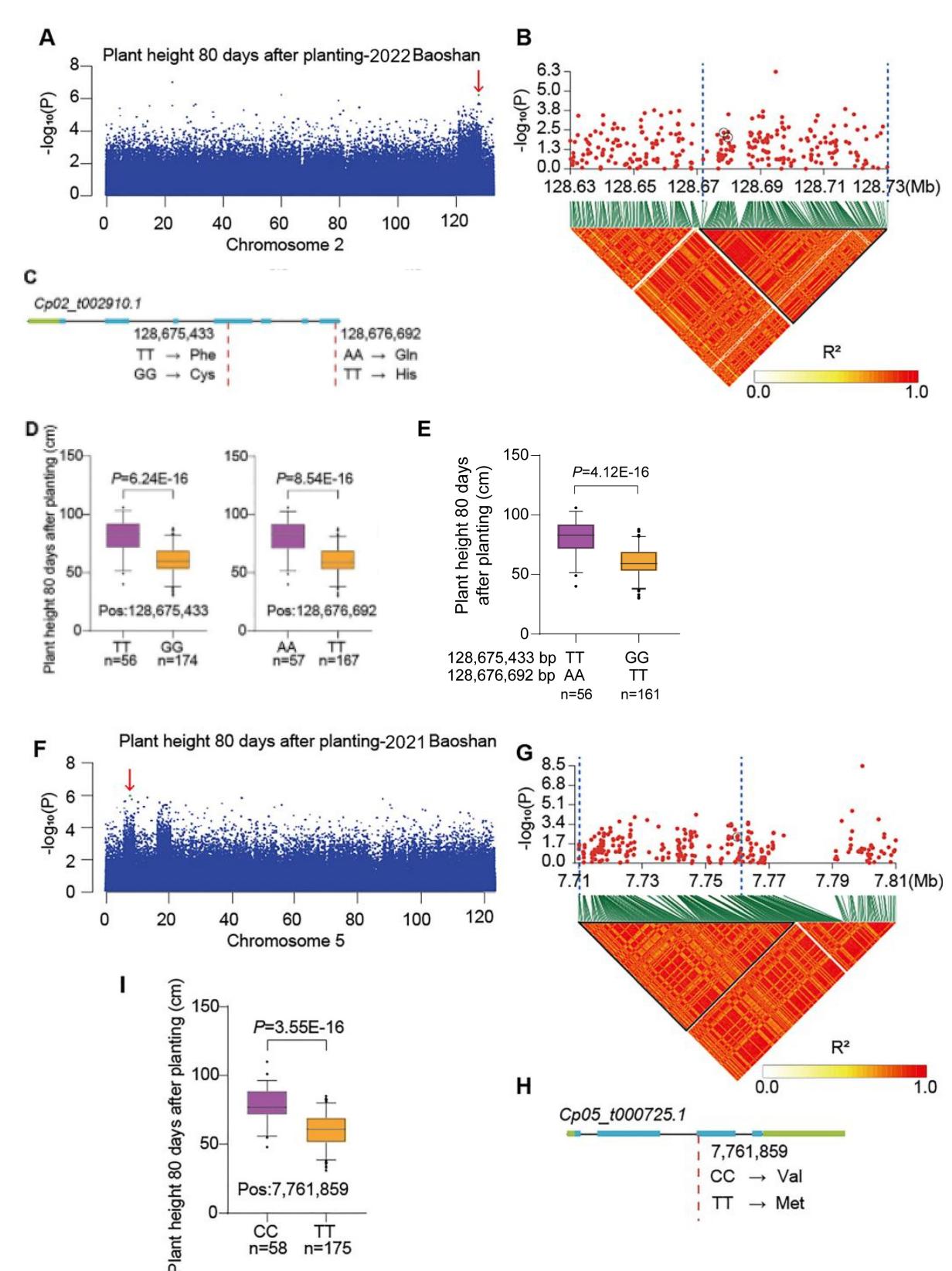


Figure S23. GWAS identification of candidate genes for plant height. (A-E) GWAS identification of *Cp02_t002910.1* as a candidate gene for plant height 80 days after planting on chromosome 2. (A) Manhattan plots for plant height 80 days after planting on chromosome 2 using GEMMA-MLM. Red arrow indicates the significant GWAS peak. (B) Local manhattan plot (top) and linkage disequilibrium heat map (bottom) surrounding the GWAS signal. Blue dashed lines represent the candidate region. The core SNPs in the candidate gene are circled. (C) *Cp02_t002910.1* gene model. The dashed red lines indicate the position of nonsynonymous SNPs. (D-E) Comparison of plant height 80 days after planting based on the core SNP variants in the candidate gene. (F-I) GWAS identification of *Cp05_t000725.1* as a candidate gene for plant height 80 days after planting on chromosome 5. For the box plots, the center line represents the median, box limits indicate the upper and lower quartiles, and whiskers denote the range of the data. The significance of difference was derived with two-tailed *t*-test.


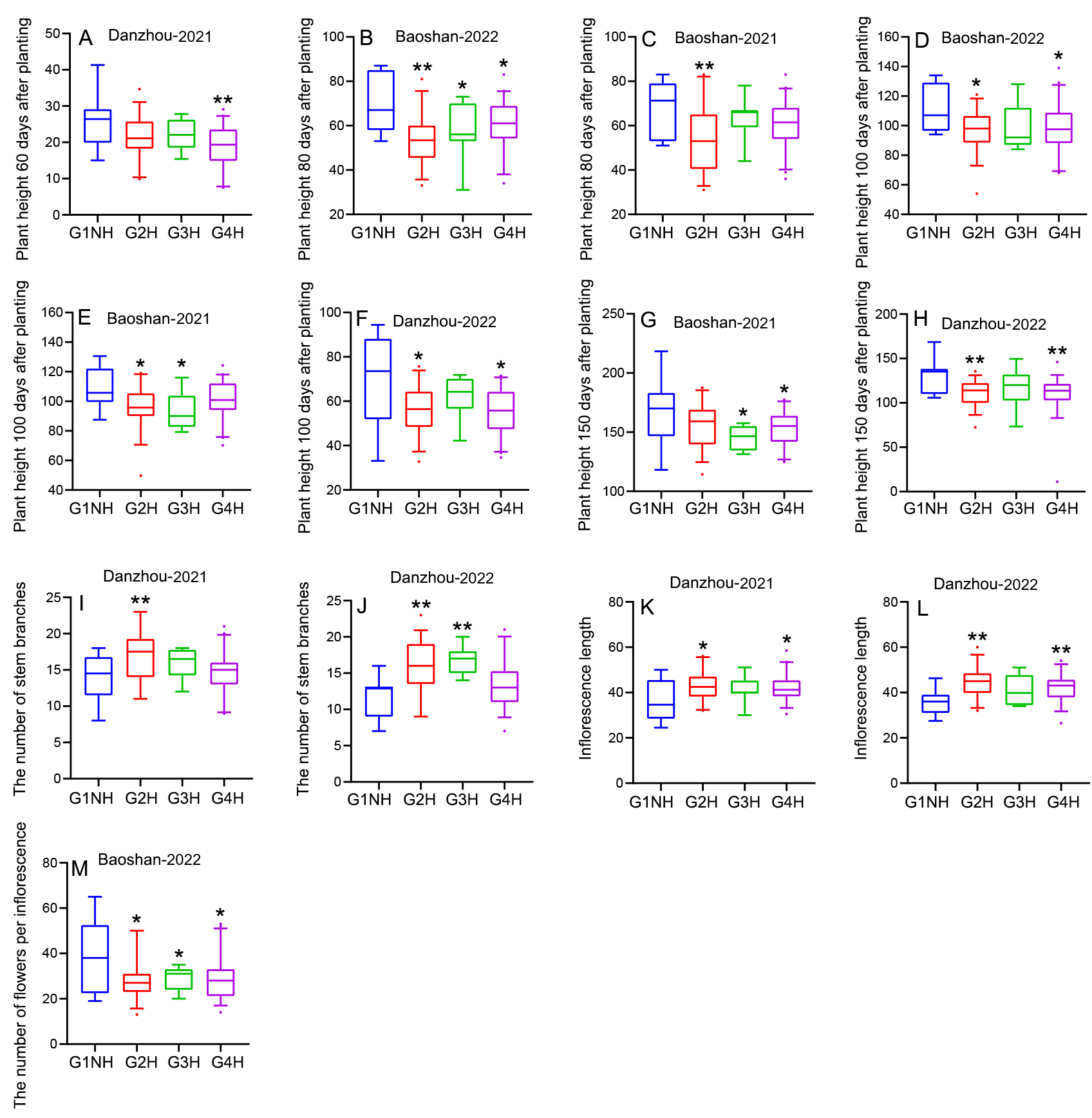


Figure S24. Significant traits divergence of plant height, the number of stem branches, inflorescence length and the number of flowers per inflorescence between G1NH and G2H/G3H/G4H. G1NH, Non-Hainan accessions in Group 1. G2H, Hainan accessions in Group 2. G3H, Hainan accessions in Group 3. G4H, Hainan accessions in Group 4. The center line represents the median, box limits indicate the upper and lower quartiles, and whiskers denote the range of the data. The significance of difference was derived with two tailed *t*-test by **P* < 0.05 or ***P* < 0.01.


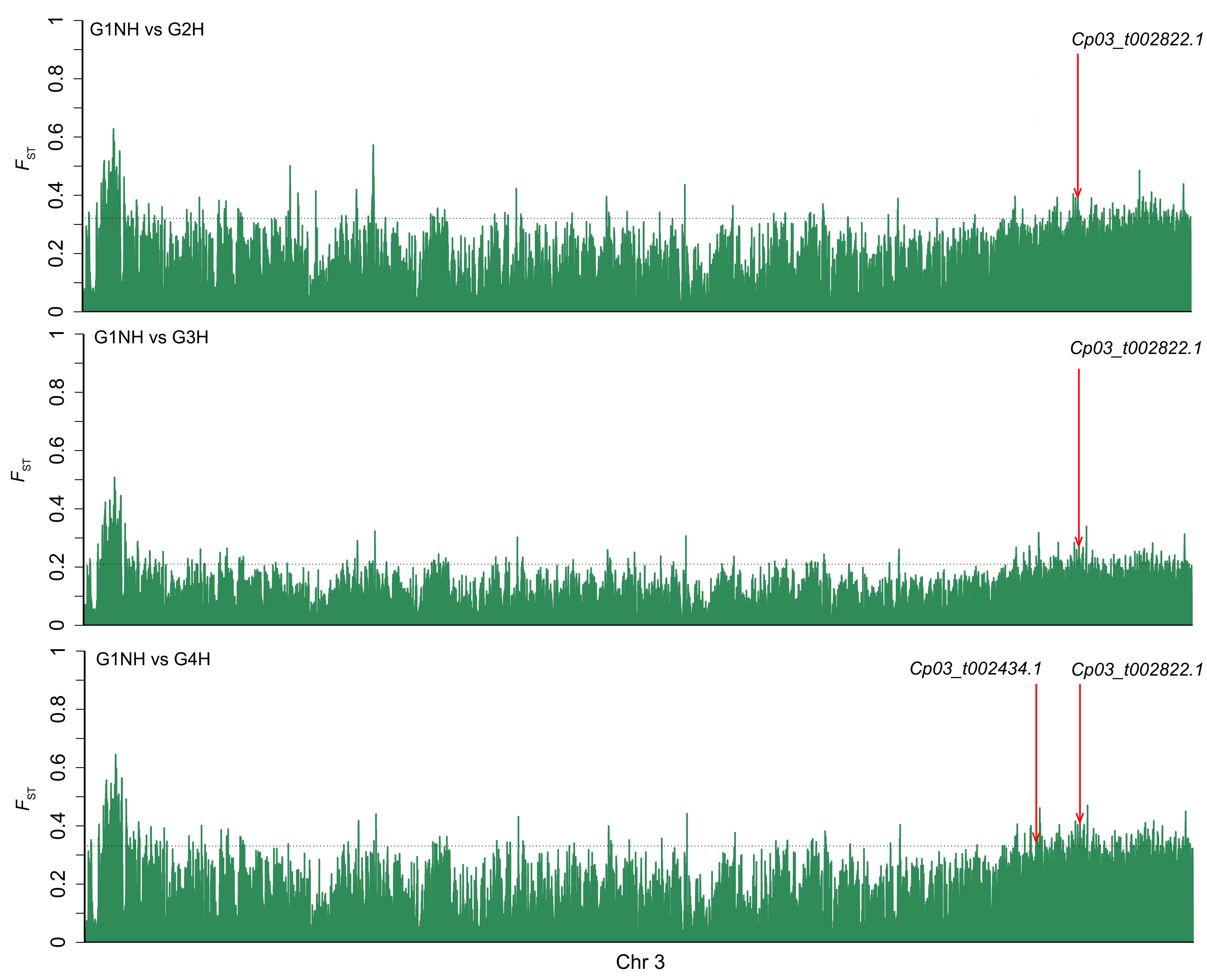


Figure S25. Divergent regions between G1NH and G2H/G3H/G4H on chromosome 3. G1NH, Non-Hainan accessions in group 1. G2H, Hainan accessions in group 2. G3H, Hainan accessions in group 3. G4H, Hainan accessions in group 4. The horizontal black dashed lines indicate values above the 95% threshold for divergence sweeps. The red arrows indicate the position of the sweeps where candidate genes are located.


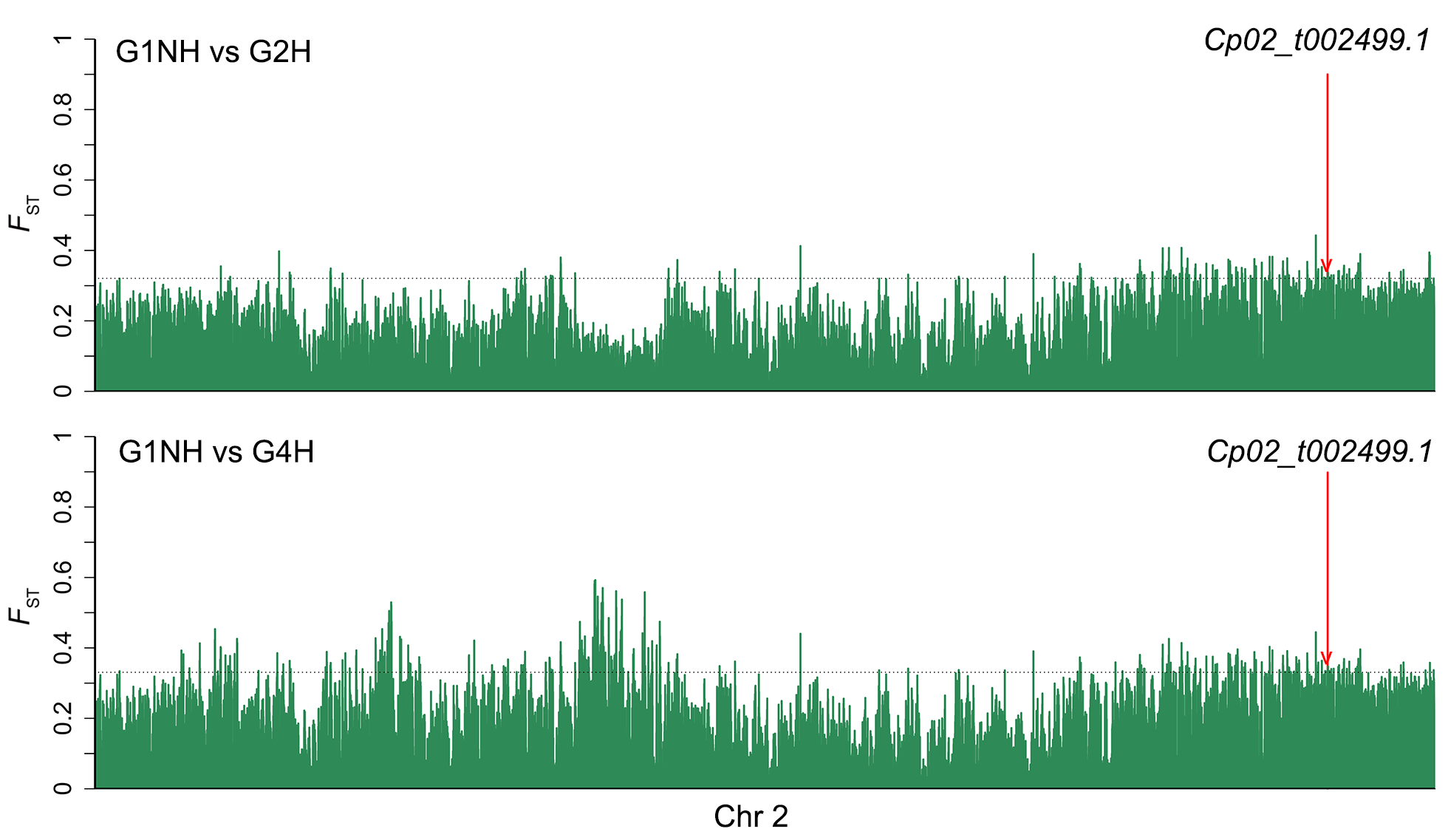


Figure S26. Divergent regions between G1NH and G2H/G4H on chromosome 2. G1NH, Non-Hainan accessions in group 1. G2H, Hainan accessions in group 2. G4H, Hainan accessions in group 4. The horizontal black dashed lines indicate values above the 95% threshold for divergence sweeps. The red arrows indicate the position of the sweeps where candidate gene is located.


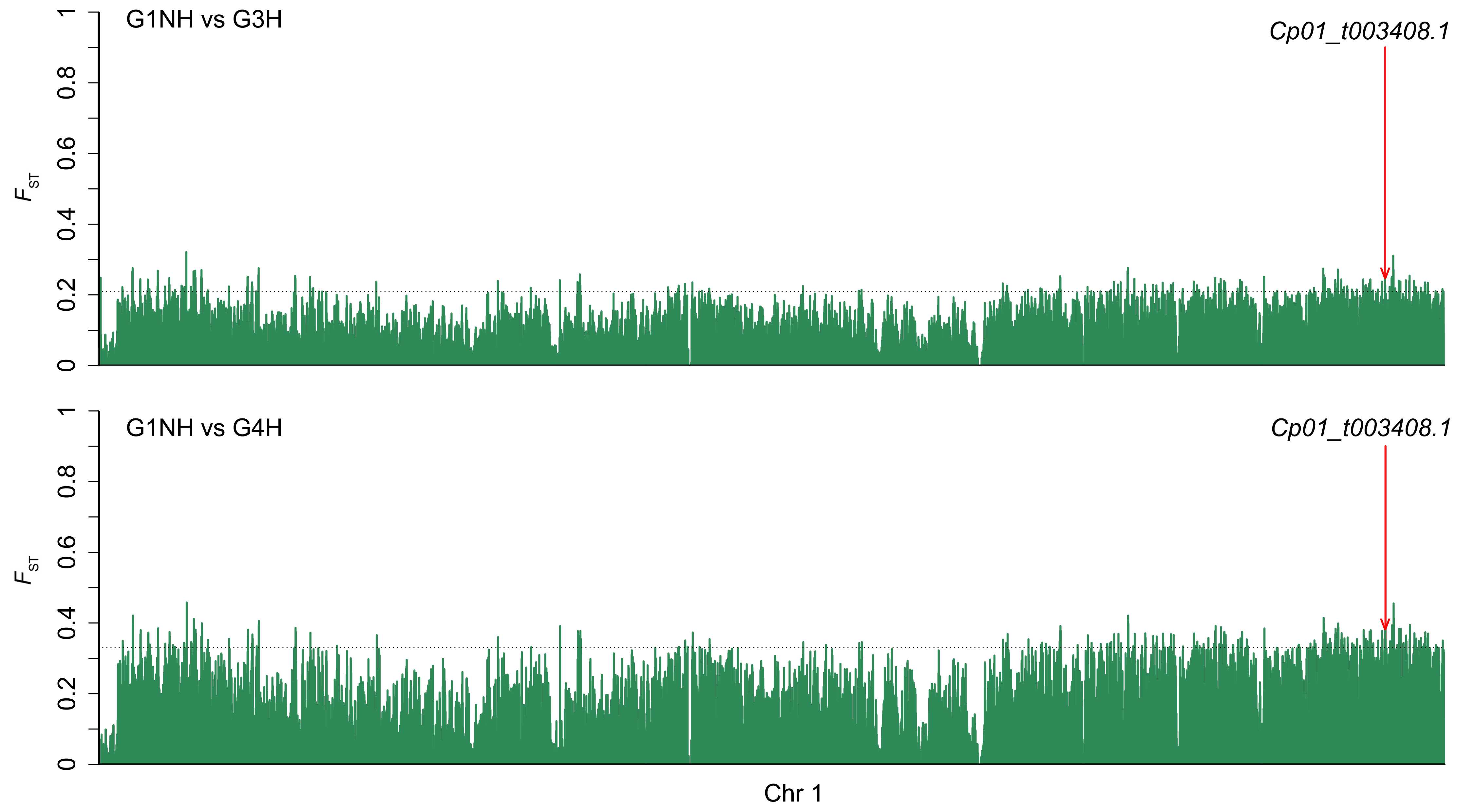


Figure S27. Divergent regions between G1NH and G3H/G4H on chromosome 1. G1NH, Non-Hainan accessions in group 1. G3H, Hainan accessions in group 3. G4H, Hainan accessions in group 4. The horizontal black dashed lines indicate values above the 95% threshold for divergence sweeps. The red arrows indicate the position of the sweeps where candidate gene is located.


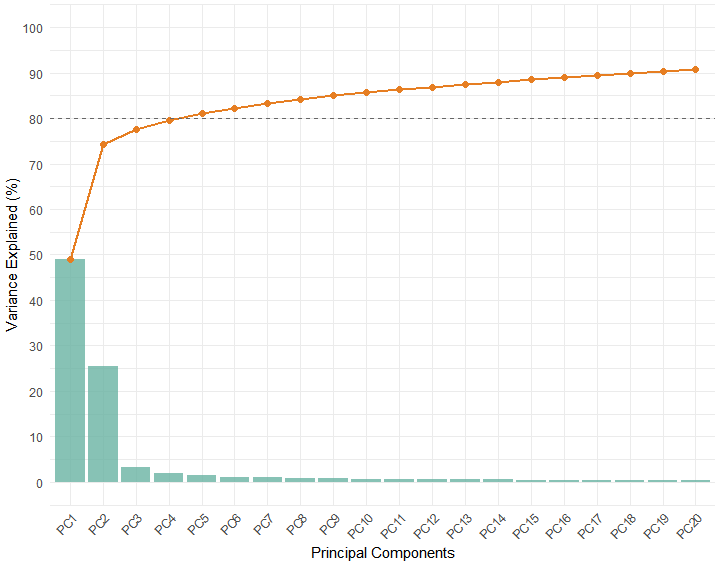


Figure S28. Scree plot of principal components (PCs) derived from the whole-genome SNP data. The bar chart represents the individual percentage of genetic variance explained by each of the first 20 principal components. The line graph illustrates the cumulative percentage of variance explained.
